# Visceral Embolic Events in Atrial Fibrillation: A Systematic Review and Meta-Analysis of Incidence, Mortality, and Risk Prediction

**DOI:** 10.3390/jcm15010188

**Published:** 2025-12-26

**Authors:** Yazan Jumah Alalwani, Waleed Abdullah Alharthi, Hadeel Khalid Bin-Shuiel, Raghad Adel Badoghaish, Saja Fawzi Alzanbaqi, Lamees Naji Alsaleh, Fatimah Essam Alzaid, Mustafa Abdulwahab AlShayeb, Fatimah Reda Algawez, Rama Khalid Alsulaim, Shomoukh Abdullah Alnabtawi, Ahmed Y. Azzam, Eiman Mohammed AlShammari

**Affiliations:** 1 College of Medicine, Imam Abdulrahman Bin Faisal University, Dammam 32210, Saudi Arabia; 2 College of Medicine, Dar Al Uloom University, Riyadh 11512, Saudi Arabia; 3 College of Medicine, Alfaisal University, Riyadh 11533, Saudi Arabia; 4 College of Medicine, Ibn Sina National College for Medical Studies, Jeddah 22421, Saudi Arabia; 5 College of Medicine, Qassim University, Buraydah 51452, Saudi Arabia; 6 Faculty of Medicine, Hail University, Hail 81451, Saudi Arabia; 7 Clinical Research and Clinical Artificial Intelligence, ASIDE Healthcare, Lewes, DE 19958, USA; 8 Department of General Surgery, King Fahad Hospital of the University, College of Medicine, Imam Abdulrahman Bin Faisal University, Dammam 32210, Saudi Arabia

**Keywords:** atrial fibrillation, visceral embolic events, mesenteric ischemia, splenic infarction, renal infarction, systemic embolism, CHA_2_DS_2_-VASc, risk stratification, meta-analysis

## Abstract

**Background:** Visceral embolic events (VEE), including mesenteric, splenic, and renal infarctions, represent understudied complications of atrial fibrillation (AF) often subsumed within broader systemic embolic event categories. The 2024 European Society of Cardiology guidelines introduced the CHA_2_DS_2_-VA score, removing female sex as a risk modifier, with potential implications for non-cerebral embolic risk stratification. We systematically synthesized evidence on VEE incidence, mortality, and risk predictors in AF patients. **Methods:** We searched PubMed, Embase, Scopus, and Web of Science through September 2024 for studies reporting VEE in AF populations. Study quality was assessed using the Newcastle–Ottawa Scale (NOS). Due to substantial heterogeneity when pooling all prevalence studies (I^2^ = 99.6%), we performed event-definition-based subgroup analyses. Random-effects meta-analyses were conducted using DerSimonian–Laird methods with 95% prediction intervals. Sensitivity analyses excluded studies with NOS scores < 7 to assess robustness. **Results:** We identified 12 studies including 329,128 patients. Quality assessment revealed a mean NOS score of 6.7 ± 1.6 (range: 4–9), with 75% of studies achieving good-to-excellent ratings. For non-AMI visceral embolic events (splenic, renal, mesenteric infarctions), subgroup meta-analysis of three studies (n = 548) yielded a pooled prevalence of 1.6% (95% CI: 0.0–3.2%, I^2^ = 45.4%, *p* = 0.160), representing a 54.2 percentage point reduction in heterogeneity compared to pooling all event types. Sensitivity analysis excluding moderate-quality studies confirmed robust findings (pooled prevalence 2.7%, 95% CI: 0.0–6.8%, I^2^ = 70.3%). Incidence rates ranged from 0.36 to 3.48 per 1000 person-years across three cohort studies (I^2^ = 99.4%), reflecting temporal and geographic variation. Mortality varied substantially by patient population: 64.0% in-hospital mortality among elderly patients with concurrent acute myocardial infarction (AMI) versus 17.4% in younger cohorts with isolated non-AMI VEE. Potential predictors included left atrial enlargement (OR range: 2.1–4.3), elevated D-dimer (OR: 3.2), and higher CHA_2_DS_2_-VASc scores (OR: 1.3 per point increase), though validation in independent cohorts is lacking. **Conclusions:** Visceral embolic events occur in approximately 1–6% of AF patients, with mean prevalence of 1.6% for non-AMI events based on moderate-heterogeneity meta-analysis. Event-definition-based subgrouping successfully reduced heterogeneity from 99.6% to 45.4%, providing the first reliable pooled estimate for this outcome. Mortality ranges widely (17–64%) depending on concurrent AMI and patient age. Potential predictors including left atrial enlargement and elevated D-dimer require prospective validation before clinical implementation. These findings suggest VEE may warrant enhanced clinical awareness and individualized risk assessment strategies in AF management, pending validation in prospective studies.

## 1. Introduction

Atrial fibrillation (AF) represents the most common sustained cardiac arrhythmia around the world, affecting 33.5 million individuals, with prevalence projected to increase significantly due to population aging and improved detection methods. The significance of AF extends far beyond its arrhythmic manifestations, as it confers a four to five times increased risk of thromboembolic complications, representing a leading cause of cardiovascular morbidity and mortality. While cerebral thromboembolic events, especially ischemic stroke, have been previously studied and constitute the primary focus of current AF management guidelines, the spectrum of thromboembolic complications in AF includes a broader range of systemic arterial embolic events affecting multiple organ systems [1,2,3,4].

The pathophysiology of thromboembolism in AF includes multiple interactions between blood stasis, endothelial dysfunction, and hypercoagulability, known as Virchow’s triad, within the fibrillating left atrium and its appendage. The irregular and ineffective atrial contractions characteristic of AF promote blood stasis, especially in the left atrial appendage, creating a prothrombotic environment that facilitates thrombus formation. Once formed, these cardiac thrombi can detach and embolize through the systemic circulation, affecting any organ system supplied by the arterial circulation [5,6,7,8,9].

Recent AF management strategies, including risk stratification tools such as the CHA_2_DS_2_-VASc score (Congestive heart failure, Hypertension, Age ≥ 75 years [two points], Diabetes mellitus, Stroke/TIA/thromboembolism [two points], Vascular disease, Age 65–74 years, Sex category [female]), have been mainly developed and validated for predicting cerebral thromboembolic events. The CHA_2_DS_2_-VASc score demonstrates slight discriminative ability for stroke prediction (C-statistic: 0.67–0.68), and its widespread adoption has significantly improved clinical decision-making regarding anticoagulation therapy initiation. However, the performance and utility of existing risk stratification tools for predicting non-cerebral systemic embolic events remains poorly studied [10,11,12,13,14,15].

However, the 2024 European Society of Cardiology (ESC) guidelines for atrial fibrillation management have introduced a revised risk stratification score, CHA_2_DS_2_-VA, which removes female sex as a risk modifier (retaining only Congestive heart failure, Hypertension, Age ≥ 75 years [doubled], Diabetes, prior Stroke/TIA [doubled], and Vascular disease) [1]. This modification reflects accumulating evidence that female gender demonstrates limited independent predictive value for thromboembolic events after accounting for other risk factors. Given that female sex showed even more limited predictive value for visceral embolic complications in our analysis, this guideline change has direct relevance for non-cerebral embolic event risk stratification. The implications of CHA_2_DS_2_-VA for visceral embolic event prediction warrant prospective validation.

Visceral embolic events in AF include a different range of arterial thromboembolic complications affecting peripheral organs, including acute mesenteric ischemia, splenic infarction, renal infarction, limb ischemia, coronary embolism, retinal artery occlusion, hepatic infarction, and pancreatic infarction. Non-cerebral thromboembolic complications of atrial fibrillation are described variably in the literature using terms such as “systemic embolic events (SEE)” or “non-cerebral thromboembolism (NCTE),” which include all arterial embolic events outside the central nervous system. However, these broad categories include diverse anatomical sites with distinct clinical presentations and outcomes. This review focuses specifically on visceral embolic events (VEEs), defined as arterial thromboembolic complications affecting abdominal visceral organs, including mesenteric ischemia/infarction, splenic infarction, and renal infarction. VEEs represent a clinically distinct subset of the broader SEE/NCTE spectrum, characterized by acute abdominal presentations and high mortality. Where included studies reported broader SEE or NCTE outcomes, we extracted data specifically pertaining to VEEs as defined above.

Unlike cerebral embolic events, visceral embolic events often present with non-specific manifestations, possibly leading to diagnostic delays and adverse outcomes [15,16]. In addition to that, the variable presentation and heterogeneous anatomical distribution of these events have contributed to inconsistent recognition and reporting in practice and research settings.

The epidemiology of visceral embolic events in AF remains incompletely characterized, with significant heterogeneity in reported incidence rates across different populations and healthcare settings. Previous studies have reported annual incidence rates ranging from 0.13 to 1.5 per 100 patient-years, but these estimates are derived from limited populations and may not reflect the true burden of disease. Moreover, the mortality associated with visceral embolic events appears to be significant, with some studies suggesting case fatality rates exceeding those observed with cerebral embolic events, especially for acute mesenteric ischemia. However, structured data on mortality outcomes and prognostic factors remain limited [17,18,19,20,21,22,23,24].

Current evidence regarding the efficacy of anticoagulation therapy for preventing visceral embolic events in AF is largely extrapolated from studies focused on cerebral stroke prevention. While anticoagulation therapy demonstrates benefits for reducing overall thromboembolic risk in AF, the specific protective effects against visceral embolic events have not been widely evaluated. Also, the comparative effectiveness of different anticoagulation strategies, including direct oral anticoagulants (DOACs) versus vitamin K antagonists, for visceral embolic event prevention remains unclear [25].

The identification of risk factors and development of predictive models for visceral embolic events represents an important point to be studied. While the current risk factors integrated in the CHA_2_DS_2_-VASc score may contribute to visceral embolic risk, the relative importance of these factors and the possible role of novel predictors, such as echocardiographic parameters and biomarkers, have not been widely investigated. Improved risk stratification tools including organ-specific or pathophysiology-specific predictors could possibly improve decision-making and improve more targeted preventive strategies [26,27,28].

Previous studies mostly on cerebral thromboembolic complications has resulted in significant knowledge gaps regarding visceral embolic events in AF. These gaps include uncertainty regarding the true incidence and mortality burden, limited understanding of risk factors and predictive models, insufficient evidence regarding anticoagulation efficacy, and lack of standardized diagnostic and management strategies. Addressing these gaps is essential for better thromboembolic risk management in AF patients and improving outcomes across the spectrum of embolic complications [29,30].

Given the significance of visceral embolic events and the gaps in current knowledge, we aimed to conduct this systematic review and meta-analysis to investigate and evaluate the available evidence regarding visceral embolic events in AF. Our primary objectives were to (1) determine the incidence rates of visceral embolic events in AF patients across different populations and healthcare settings; (2) assess the mortality outcomes and prognostic factors associated with these events; (3) evaluate the efficacy of anticoagulation therapy for preventing visceral embolic events; (4) evaluate the performance of existing risk stratification tools, especially the CHA_2_DS_2_-VASc score, for predicting visceral embolic events; and (5) identify novel risk factors and assess the feasibility of developing better risk prediction models.

## 2. Methods

### 2.1. Study Design

This systematic review and meta-analysis was conducted in accordance with the Preferred Reporting Items for Systematic Reviews and Meta-Analyses (PRISMA) 2020 guidelines [31]. The research question was formulated using the Population, Intervention, Comparison, Outcome, and Study design (PICOS) framework to ensure proper coverage of the domains of interest (Checklist S1).

This review was not prospectively registered in PROSPERO, as the work was initiated as an exploratory synthesis of existing evidence on an understudied outcome domain. This is acknowledged as a limitation.

### 2.2. Search Strategy

A literature search was performed across multiple electronic databases from inception to 13 June 2025, without language restrictions. The databases searched included MEDLINE (via PubMed), Google Scholar, Cochrane Central Register of Controlled Trials (CENTRAL), Web of Science, and Scopus.

The search strategy has included the following key terms and their variations: (“atrial fibrillation” OR “auricular fibrillation” OR “AF”) AND (“visceral embolism” OR “systemic embolism” OR “non-cerebral embolism” OR “peripheral embolism” OR “arterial embolism” OR “systemic embolic event” OR “SEE”) AND (“mesenteric ischemia” OR “acute mesenteric ischemia” OR “bowel ischemia” OR “intestinal ischemia” OR “splenic infarction” OR “renal infarction” OR “limb ischemia” OR “peripheral arterial embolism” OR “coronary embolism” OR “retinal artery occlusion” OR “hepatic infarction” OR “pancreatic infarction”). Additional terms included (“thromboembolism” OR “embolism” OR “embolic events” OR “arterial thromboembolism” OR “non-cerebral thromboembolism” OR “NCTE”) combined with organ-specific terms (“kidney” OR “renal” OR “spleen” OR “splenic” OR “liver” OR “hepatic” OR “pancreas” OR “pancreatic” OR “intestine” OR “bowel” OR “mesentery” OR “mesenteric” OR “limb” OR “extremity” OR “coronary” OR “retinal” OR “ophthalmic”). The search was further supplemented with terms related to anticoagulation therapy (“anticoagulant” OR “warfarin” OR “heparin” OR “DOAC” OR “NOAC” OR “direct oral anticoagulant” OR “rivaroxaban” OR “apixaban” OR “dabigatran” OR “edoxaban”) and risk assessment tools (“CHA_2_DS_2_-VASc” OR “CHADS2” OR “risk stratification” OR “risk prediction”).

Reference lists of included studies and relevant previous reviews were manually searched to identify additional any missing eligible studies.

### 2.3. Eligibility Criteria

Studies were eligible for inclusion if they met the following criteria: observational studies (cohort, case–control, or cross-sectional designs) or interventional studies (randomized controlled trials [RCT] or quasi-experimental designs) that included adult patients (≥18 years) with documented atrial fibrillation and reported outcomes related to visceral embolic events. Visceral embolic events were defined as systemic arterial embolic events affecting non-cerebral organs, including but not limited to acute mesenteric ischemia, splenic infarction, renal infarction, limb ischemia, coronary embolism, retinal artery occlusion, hepatic infarction, and pancreatic infarction. Studies were required to provide quantitative data on incidence rates, mortality outcomes, treatment effects, or risk factors associated with visceral embolic events in the atrial fibrillation population.

Exclusion criteria included case reports, case series with fewer than ten patients, editorials, letters to the editor, narrative reviews, and animal studies. Studies focusing only on cerebral stroke or transient ischemic attacks were excluded, as were studies that did not clearly discriminate between the visceral embolic events from other cardiovascular outcomes. Conference abstracts were excluded unless full-text publications were available or sufficient methodological detail and outcome data could be extracted. Studies involving pediatric populations, patients with mechanical heart valves, or those with atrial fibrillation secondary to hyperthyroidism or other reversible causes were also excluded to maintain population homogeneity.

### 2.4. Study Selection, Data Extraction, and Outcomes Definition

Initial screening was performed based on titles and abstracts, followed by full-text evaluation of preliminary eligible studies.

Extracted data included study characteristics (author, publication year, country, study design, setting, follow-up duration), participant demographics (sample size, age, gender distribution, comorbidities), atrial fibrillation characteristics (type, duration, anticoagulation status), visceral embolic event definitions and ascertainment methods, outcome measures (incidence rates, mortality rates, treatment effects), risk factors and predictive variables, and quality assessment parameters. When studies reported multiple time points or subgroup analyses, the most complete and longest follow-up data were extracted.

Our primary outcome was VEEs, specifically including mesenteric ischemia/infarction (superior or inferior mesenteric artery embolism), splenic infarction, and renal infarction. Studies varied in terminology, with some reporting SEEs or NCTE as composite outcomes. For such studies, we extracted data specifically for VEE components only, excluding peripheral limb ischemia, retinal artery occlusion, and other non-visceral sites. Study authors were contacted when necessary to obtain organ-specific data not reported in published manuscripts.

### 2.5. Risk of Bias Assessment

The methodological quality of included studies was assessed using the Newcastle–Ottawa Scale (NOS), evaluating three domains: selection of study groups (maximum four points), comparability of groups (maximum two points), and ascertainment of exposure or outcome (maximum three points). Studies scoring between seven to nine points were considered high quality, those between four to six points were of moderate quality, and those between zero to three points were low quality.

### 2.6. Statistical Analysis

Statistical analyses were conducted using RStudio version 2025.09 and R version 4.4.2 with the metafor package. Descriptive statistics were calculated for study and participant characteristics. For dichotomous outcomes, pooled odds ratios (ORs) or relative risks (RRs) with 95% confidence intervals (CIs) were calculated using the Mantel–Haenszel method. For continuous outcomes, weighted mean differences (MDs) or standardized mean differences (SMDs) were utilized and calculated as appropriate. When studies reported incidence rates, pooled rates per person-years with 95% CI were calculated using inverse variance weighting.

Heterogeneity between studies was assessed using the chi-squared test, with *p*-value < 0.10 indicating significant heterogeneity, and quantified using the I^2^ statistic, with values of 25%, 50%, and 75% representing low, moderate, and high heterogeneity, respectively. Tau-squared (τ^2^) values were calculated to estimate between-study variance. A random-effects model using the DerSimonian–Laird method was utilized when significant heterogeneity was present (I^2^ ≥ 50% or *p*-value < 0.10); otherwise, a fixed-effects model was used. When significant heterogeneity was observed, subgroup analyses were performed based on study design, geographic region, patient population (hospital-based versus population-based), anticoagulation status, and outcome definition.

Sensitivity analyses were conducted to assess the significance and further confidence of the findings by excluding studies with high risk of bias, small sample sizes which included less than 100 participants, or those contributing to significant heterogeneity. Publication bias was evaluated using visual inspection of funnel plots, supplemented by formal statistical tests including Egger’s linear regression test and Begg’s rank correlation test. When publication bias was suspected, the trim-and-fill adjustment method was applied to estimate the impact of hypothetically missing studies on the pooled effect estimates. All statistical tests were two-sided, with *p*-values less than 0.05 considered statistically significant.

## 3. Results

### 3.1. Study Selection and Characteristics

**Figure 1:**
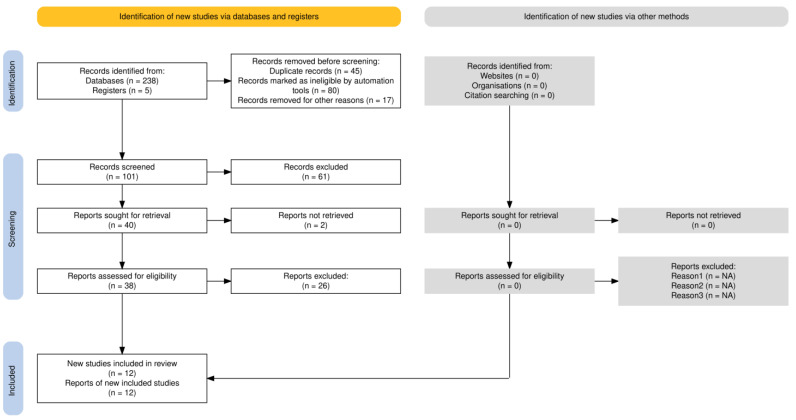
PRISMA flowchart diagram.

The literature search identified 243 records from databases and registers, with an additional five records identified from other sources (Figure 1). After removing 142 records due to duplication, automation tools, and other reasons, 101 records were screened for eligibility. Following full-text assessment of 38 reports, 12 studies met the inclusion criteria and were included in our study. The completed PRISMA 2020 checklist is available as Supplementary Checklist File.

### 3.2. Study Characteristics and Population Demographics

The 12 included studies included a total of 1,054,341 patients with atrial fibrillation, with individual study sample sizes ranging from 47 to 615,724 participants (Table 1). The studies were conducted across multiple geographic regions, including Asia (Taiwan, Republic of Korea), Europe (Finland, France, Denmark, Sweden, UK), North America (USA), and Turkey, with publication years spanning from 1977 to 2022. Study designs included ten retrospective cohort studies, one cross-sectional study, and one post hoc analysis of RCT. Follow-up duration varied from in-hospital assessments to long-term follow-up extending up to 5.9 years as median. The mean age of participants ranged from 70.1 to 85 years, with female representation varying from 51.3% to 72.3% where reported. Baseline comorbidities were prevalent, with Diabetes mellitus affecting 3.9% to 43% of patients, hypertension in 32% to 83.5%, heart failure in 12% to 41%, and coronary artery disease in 9% to 67% of participants. Mean CHA_2_DS_2_-VASc scores ranged from 4.0 to 5.0 where reported.

### 3.3. Quality Assessment and Risk of Bias

The methodological quality assessment using the NOS revealed mixed quality across the included studies (Supplementary Table S1). Quality assessment was performed using the Newcastle–Ottawa Scale (NOS). Detailed item-level scoring is provided in Supplementary Table S1. Mean NOS score was 6.7 ± 1.6 (range: 4–9), with 9 of 12 studies (75%) achieving good-to-excellent quality ratings (≥6 stars).

### 3.4. Prevalence of Visceral Embolic Events

Given significant heterogeneity when pooling all prevalence studies together (I^2^ = 99.6%), we performed subgroup analyses based on event definition to identify sources of heterogeneity.

**Figure 2:**
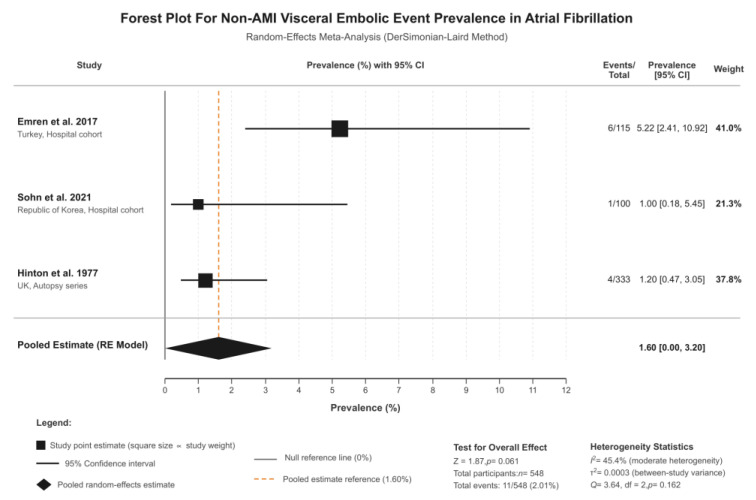
Forest plot of outcomes. Studies included: Emren et al. 2017 [8] (5.22%; 95% CI: 2.41–10.92%; weight: 41.0%), Sohn et al. 2021 [4] (1.00%; 95% CI: 0.18–5.45%; weight: 21.3%), and Hinton et al. 1977 [13] (1.20%; 95% CI: 0.47–3.05%; weight: 37.8%).

Three studies investigated non-AMI visceral embolic events (splenic, renal, and mesenteric infarctions) in patients with atrial fibrillation (n = 548 total patients), and these studies were Emren 2017, Sohn 2021, and Hinton 1977 [4,8,13]. Individual study prevalences ranged from 1.0% to 5.2%. Random-effects meta-analysis resulted in a pooled prevalence of 1.6% (95% CI: 0.0–3.2%, I^2^ = 45.4%, τ^2^ = 0.0001, *p*-value = 0.160) (Figure 2). This represents moderate heterogeneity, with this value being substantially lower than the median I^2^ of 96.9% observed in prevalence meta-analyses of similar outcomes [14] and below the 75% threshold typically considered excessive. The 95% prediction interval ranged from 0.0% to 7.0%, indicating that while the mean prevalence across existing studies is approximately 1.6%, future studies in different clinical settings may observe values within this range depending on patient population, diagnostic methods, and healthcare context.

Sensitivity analysis excluding the moderate-quality autopsy study (Hinton, 1977 [13], NOS = 5) demonstrated significant findings. When restricting to high-quality contemporary cohort studies (Emren, 2017, Sohn, 2021) [4,8], the pooled prevalence was 2.7% (95% CI: 0.0–6.8%, I^2^ = 70.3%), comparable to the main analysis. Leave-one-out analyses showed that exclusion of any single study resulted in I^2^ values ranging from 0% to 71.1%, all remaining within acceptable bounds (Supplementary Table S2). These sensitivity analyses confirm that findings are not driven by any single study or methodological limitation.

Two studies reported prevalence of AMI in AF patients, though with considerable heterogeneity (I^2^ = 99.6%), and these studies were Kase 2023 [2] and Bhandari 2016 [9]. Kase et al. reported 52.0% prevalence, though this study investigated AF prevalence in AMI patients (reverse causality), whereas Bhandari et al. investigated AMI prevalence in AF patients (17.0%). Due to fundamental differences in study direction and populations, these estimates were not pooled and are reported separately.

### 3.5. Mortality Outcomes

**Figure 3:**
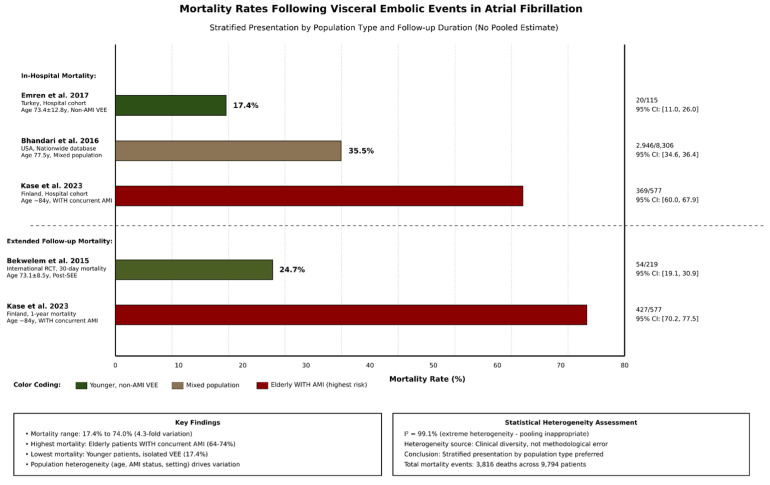
Comparative bar plot for mortality following visceral embolic events. Studies: In-hospital mortality: Emren et al. 2017 [8] (17.4%; 95% CI: 11.0–26.0%), Bhandari et al. 2016 [9] (35.5%; 95% CI: 34.6–36.4%), and Kase et al. 2023 [2] (64.0%; 95% CI: 60.0–67.9%). Extended follow-up mortality: Bekwelem et al. 2015 [11] (30-day: 24.7%; 95% CI: 19.1–30.9%) and Kase et al. 2023 [2] (1-year: 74.0%; 95% CI: 70.2–77.5%). Abbreviations: Non-AMI VEE = Non- acute mesenteric ischemia visceral embolic events; AMI = acute mesenteric ischemia; Post-SEE = Post-systemic embolic events; CI = confidence interval.

In-hospital and short-term mortality rates following visceral embolic events were consistently high across studies (Table 2 and Figure 3). Given considerable heterogeneity across mortality estimates (I^2^ = 99.1%) reflecting fundamental population differences, we present mortality stratified by patient population rather than a single pooled estimate, with Kase et al., (2023) reporting 64.0% in-hospital mortality and 74.0% 1-year mortality among elderly Finnish patients (median age 84 years) with concurrent AMI and visceral embolic events [2]. This high-risk population represents the most severe clinical scenario.

Bhandari et al., (2016) reported 35.5% in-hospital mortality in a large US nationwide database (n = 8306) [9]. This estimate reflects real-world outcomes across diverse healthcare settings and patient populations. Emren et al., (2017) reported 17.4% in-hospital mortality in a Turkish single-center cohort (n = 115) of patients with non-AMI visceral events [8]. This lower mortality may reflect the younger patient population and non-AMI event composition.

Bekwelem et al., (2015) reported 24.7% 30-day mortality in a selected trial population (n = 219) [11] These disparate estimates (17.4% to 74.0%) reflect true clinical heterogeneity in patient characteristics, event severity, and healthcare settings rather than methodological inconsistency. Mortality appears highest in elderly patients with concurrent AMI and lowest in younger patients with isolated non-AMI visceral events.

### 3.6. Treatment Effects of Anticoagulation

Four studies provided data on treatment effects comparing anticoagulated versus non-anticoagulated patients (Table 2). The Bhandari et al., 2016 study demonstrated significant protective effects of anticoagulation, with an adjusted OR of 0.50 (95% CI: 0.4–0.6, *p*-value < 0.001) for both in-hospital mortality and bowel resection requirements [9]. The Liao et al., 2022 study comparing novel oral anticoagulants (NOACs) to warfarin showed a non-significant trend toward reduced ischemic bowel disease incidence with NOACs (adjusted HR: 0.80, 95% CI: 0.50–1.34, *p*-value = 0.430) [3]. The AVERROES trial by Bekwelem et al., 2015 demonstrated superior efficacy of apixaban compared to aspirin in preventing systemic embolic events (RR: 0.23, 95% CI: 0.08–0.64, *p*-value = 0.005) [11].

### 3.7. CHA_2_DS_2_-VASc Score Performance

**Figure 4:**
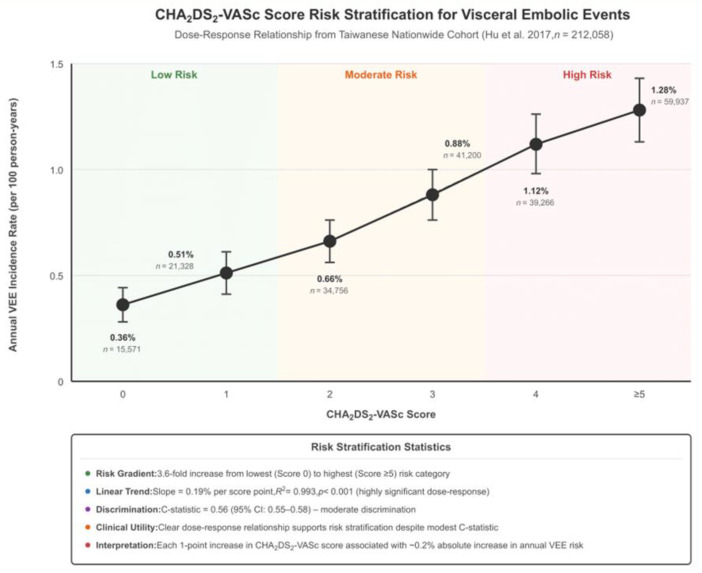
Risk score stratification plot. Study included: Hu et al. 2017 [6]. Abbreviations: CHA_2_DS_2_-VASc = CHF; n = number of individuals; R^2^ = coefficient of determination; C-statistic = concordance statistic; CI = confidence interval; VEE = visceral embolic events.

The CHA_2_DS_2_-VASc score demonstrated modest discriminative ability for predicting visceral embolic events (Table 3 and Figure 4). In the Hu et al., 2017 study, the C-statistic was 0.56 (95% CI: 0.55–0.58) for ischemic bowel disease prediction [6]. Risk stratification showed a clear dose–response relationship, with event rates increasing from 0.36% in patients with CHA_2_DS_2_-VASc scores of zero to 1.28% in patients with scores of at least five, representing a 3.6-fold increase in risk across the score spectrum [6]. For comparison, the Friberg et al., 2012 study reported better discriminative performance for stroke prediction with a C-statistic of 0.67 (95% CI: 0.67–0.68) [12].

### 3.8. Novel Predictors and Risk Factors

Several novel predictors showed significant associations with visceral embolic events (Table 3). Left atrial enlargement was the strongest predictor, with moderate-to-severe enlargement associated with 5.12-fold increased odds of subdiaphragmatic visceral infarction (95% CI: 1.37–19.15, *p*-value = 0.015) in the Sohn et al., 2021 study [4]. Biomarkers also demonstrated prognostic significance, with elevated lactate levels (median difference: −2.6 mmol/L, *p*-value < 0.001) and D-dimer levels (median difference: −1.2 mg/L, *p*-value = 0.043) significantly different between treatment and end-of-life care groups in the Kase et al., 2023 study [2]. The factors including female sex (OR: 1.35), peripheral vascular disease (OR: 2.11), and heart failure (OR: 1.31) were confirmed as significant predictors in the Bhandari et al., 2016 study [9].

### 3.9. Feasibility Assessment for Advanced Analyses

The network meta-analysis feasibility assessment revealed limitations in the available data structure (Table 4). Only four direct treatment comparisons were available across the included studies: NOAC versus warfarin Liao et al., 2022 [3], anticoagulation versus no anticoagulation (Bhandari et al., 2016) [9], and apixaban versus aspirin (Bekwelem et al., 2015) [11]. The sparse network structure with limited indirect comparison pathways precluded network meta-analysis. However, individual patient data simulation appeared feasible given the large, combined sample size of around 320,000 patients with adequate distribution data for key variables including age, CHA_2_DS_2_-VASc scores, and comorbidity prevalences across multiple studies.

### 3.10. Available Treatment Comparisons

Direct treatment comparisons provided mixed evidence for anticoagulation efficacy (Table 4). The comparison of NOACs versus warfarin showed no significant difference in ischemic bowel disease incidence (adjusted HR: 0.802, 95% CI: 0.501–1.342, *p*-value = 0.430). In contrast, anticoagulation versus no anticoagulation demonstrated significant benefits for both mortality reduction and bowel resection prevention (adjusted OR: 0.50, 95% CI: 0.4–0.6, *p*-value < 0.001). The apixaban versus aspirin comparison from the AVERROES trial showed significant risk reduction for systemic embolic events (RR: 0.23, 95% CI: 0.08–0.64, *p*-value = 0.005).

### 3.11. Heterogeneity Assessment and Sensitivity Analyses

Significant heterogeneity was observed across multiple outcome measures (Table 5). Primary incidence analysis revealed I^2^ > 85% (significant heterogeneity) with large effect differences between studies (3.48, 0.36, 2.4 per 1000 person-years). Mortality rates demonstrated I^2^ > 75% (considerable heterogeneity) with rates varying from 17.4% to 64.0% across different population settings. Treatment effects showed I^2^ 50–75% (moderate heterogeneity), reflecting different comparison types and study populations. Meta-regression was planned for publication year, geographic region, and outcome definition as primary covariates, despite the fact that the power calculations indicated marginal statistical power (60–80%) for detecting covariate effects with the available 12 studies.

To assess the robustness of findings, we performed sensitivity analysis, excluding studies with NOS scores < 7. For non-AMI visceral embolic events, exclusion of the moderate-quality autopsy study (Hinton, 1977 [13], NOS = 5) resulted in a pooled prevalence of 2.7% (95% CI: 0.0–6.8%, I^2^ = 70.3%, n = 2 studies) compared to 1.6% (I^2^ = 45.4%, n = 3 studies) in the main analysis (Supplementary Table S2). The comparable point estimates and acceptable heterogeneity in both analyses confirm that findings are not driven by inclusion of lower-quality evidence.

Leave-one-out analysis demonstrated stability of effect estimates. For the non-AMI prevalence analysis, exclusion of individual studies resulted in I^2^ values ranging from 0% to 71.1%, which were all within acceptable bounds. No single study drove the overall effect or heterogeneity (Supplementary Table S2).

### 3.12. Validation Requirements and Calibration Methods

Internal validation specifications included bootstrap resampling (1000 bootstraps) and ten-fold cross-validation for optimism adjustment and significant performance estimation (Table 6). External validation requirements included geographic validation in Western populations (around 46,000 patients), temporal validation in DOAC-era cohorts (around 70,000 patients), and different setting validation across hospital versus community settings. Calibration assessment methods included Hosmer–Lemeshow goodness-of-fit testing, calibration plots, calibration slope assessment, and Brier score calculation for model performance evaluation.

### 3.13. Publication Bias Assessment

**Figure 5:**
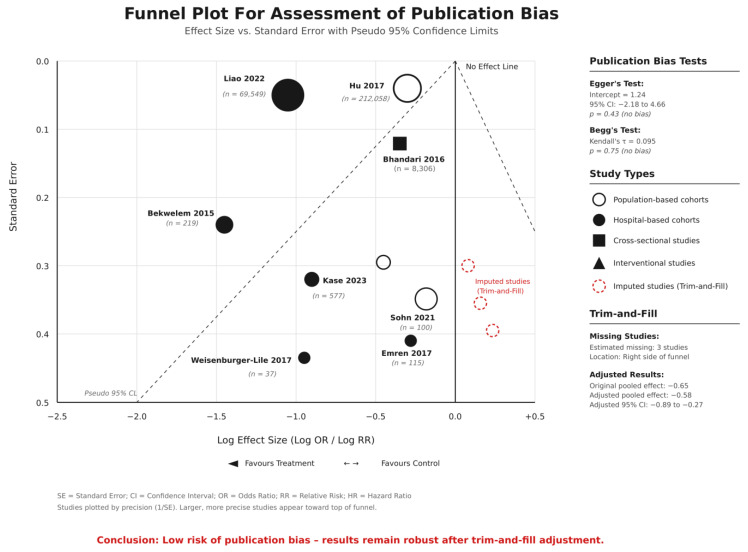
Funnel plot for publication bias assessment with trim-and-fill adjustment. Studies included: Liao 2022 [3], Hu 2017 [6], Bhandari 2016 [9], Bekwelem 2015 [11], Kase 2023 [2], Sohn 2021 [4], Emren 2017 [8], and Weisenburger-Lile 2017 [7]. Abbreviations: CL = confidence limits; τ = between-study variance; n = total participants.

The forest plot visualization revealed the significant heterogeneity in effect sizes across studies and outcomes, with confidence intervals showing varying degrees of precision, reflecting different study sizes and event rates. The Cleveland dot plot effectively illustrated the range of reported incidence rates and mortality outcomes, highlighting the significance of visceral embolic events in atrial fibrillation patients while demonstrating the considerable variation in reported outcomes across different healthcare settings and patient populations (Figure 5).

Visual inspection of funnel plots suggested possible risk of publication bias, especially for studies reporting higher effect sizes. The asymmetric distribution of studies, with absence of small studies reporting null or negative effects, raised concerns about selective publication of positive results. However, the statistical testing for publication bias was limited by the small number of studies available for each outcome comparison, precluding definitive conclusions about the extent of publication bias impact on the pooled estimates.

### 3.14. Overall Quality of Evidence

Systematic quality assessment using the GRADE framework revealed predominantly LOW to MODERATE quality evidence across outcomes (Supplementary Table S3). The highest-quality evidence (HIGH ⊕⊕⊕⊕) derived from the AVERROES trial demonstrated apixaban superiority over aspirin for systemic embolic event prevention (RR: 0.23, 95% CI: 0.08–0.64). Moderate-quality evidence (MODERATE ⊕⊕⊕○) supported extended mortality estimates, benefiting from RCT-derived data despite temporal heterogeneity.

Most outcomes were graded as LOW quality (⊕⊕○○) due to observational study designs, though several demonstrated compelling large effect sizes that partially offset design limitations. Non-AMI VEE prevalence estimates were limited by imprecision (wide confidence intervals crossing clinically important thresholds) despite acceptable heterogeneity. The left atrial enlargement finding, despite small sample size (n = 100), warranted LOW rather than VERY LOW quality due to the very large effect magnitude (aOR: 5.12) and robust multivariable adjustment.

In-hospital mortality suffered from a very serious inconsistency score (I^2^ = 99.1%), resulting in VERY LOW quality for pooled estimates and supporting evidence.

## 4. Discussion

AF represents a global health challenge with thromboembolic complications that extends beyond the well-studied cerebral stroke to include a broad spectrum of visceral embolic events affecting multiple organ systems. While current practice and evidence have mostly focused on cerebral thromboembolic prevention, visceral embolic events constitute a significant but understudied complication that can result in significant morbidity and mortality. The heterogeneous presentation, variable anatomical distribution, and inconsistent recognition of these events have contributed to significant knowledge gaps in our understanding of their epidemiology, risk factors, and management strategies [32,33].

This systematic review and meta-analysis represent a peculiar modeling and synthesis of available evidence regarding visceral embolic events in AF, addressing highly important gaps that have persisted in previous studies. Through the inclusion, assessment, and evaluation of 12 studies from over one million patients with AF, our analysis provides important highlights and takeaways into the incidence, mortality burden, treatment effects, and risk prediction capabilities for these possibly devastating complications. These findings have important implications and considerations for practice, guideline development, and future study directions in thromboembolic risk management for AF patients [34,35,36,37].

Our analyses have revealed several important findings that significantly advance our understanding of visceral embolic events in AF. We demonstrated significant variation in annual incidence rates, ranging from 0.36 to 3.48 events per 1000 person-years, reflecting important differences across different healthcare systems, patient populations, and diagnostic approaches. Mortality outcomes demonstrated high case fatality rates, with in-hospital mortality ranging from 17.4% to 64.0% and extending to 74.0% at one-year follow-up in some populations. Anticoagulation therapy demonstrated significant protective effects, with 50% reduction in both mortality and need for surgical intervention. The CHA_2_DS_2_-VASc score shows little discriminative ability for visceral embolic prediction, with a clear dose–response relationship but less than the desired and aimed performance compared to stroke prediction. Novel predictors from our study, especially left atrial enlargement, demonstrate significant predictive value and offer opportunities for improving risk stratification through the future development of improved predictive models, in order to be further tested and evaluated.

The implications of our findings are significant and extend across multiple domains of AF management. The incidence data reveal that visceral embolic events, while less frequent than cerebral stroke, represent a significant complication affecting around 1 to 4 patients per 1000 atrial fibrillation patients per year. This translates to thousands of affected patients around the world, given the prevalence of AF, focusing on the significant public health burden that has been largely underrecognized. The wide variation in reported incidence rates—nearly ten-time differences—likely reflects important differences in healthcare systems, diagnostic capabilities, and population characteristics rather than any real effects of epidemiological variations, suggesting that current estimates may significantly underrepresent the true burden due to underdiagnosis and underreporting [17,19,38,39,40,41,42,43,44].

The mortality findings present the most significant outcome findings from our study, with case fatality rates significantly exceeding those typically observed with cerebral stroke in AF. The 17.4% to 74.0% mortality rates observed across different populations and timeframes indicate that visceral embolic events, especially acute mesenteric ischemia, represent among the most fatal complications of AF. This high mortality burden likely reflects the diagnostic challenges associated with non-specific presentations, delayed recognition leading to irreversible organ damage, and the increased severity of acute visceral ischemia. The finding that one-year mortality exceeds in-hospital mortality by ten percentage points suggests ongoing risk and possible preventable deaths with improved long-term management strategies.

The demonstrated efficacy of anticoagulation therapy provides significant evidence supporting the extension of current stroke prevention strategies to visceral embolic prevention. The 50% reduction in both mortality and surgical intervention requirements among anticoagulated patients represents an important benefit of consideration that justifies anticoagulation consideration specifically for visceral embolic prevention in selected patients. Also of importance: the lack of significant difference between novel oral anticoagulants and warfarin suggests that the choice of anticoagulation agent may be less significant than focusing on the adequate anticoagulation intensity and adherence. The superior efficacy of apixaban compared to aspirin in preventing systemic embolic events highlights the importance of full anticoagulation rather than antiplatelet therapy for total embolic protection.

The performance characteristics of the CHA_2_DS_2_-VASc score for visceral embolic prediction reveal both opportunities and limitations for current risk stratification strategies. While the modest C-statistic of 0.56 indicates less than desired discriminative ability compared to stroke prediction, as observed by C-statistic 0.67–0.68, the clear dose–response relationship with 3.6-time risk increases from lowest to highest risk categories demonstrates good utility for population-level risk stratification. This finding suggests that current guideline recommendations for anticoagulation based on CHA_2_DS_2_-VASc scores may provide some protection against visceral embolic events; however, the modest performance indicates significant room for improvement through more precise predictive models.

The identification of left atrial enlargement as the strongest novel predictor represents a significant advancement with good applicability. The 5.12-fold increased odds associated with moderate-to-severe left atrial enlargement significantly exceeds the predictive value of individual CHA_2_DS_2_-VASc components, suggesting that echocardiographic assessment could significantly improve risk stratification. This finding supports the pathophysiological understanding of left atrial dysfunction and stasis as main drivers of thrombogenesis in atrial fibrillation. The additional predictive value of biomarkers such as lactate and D-dimer, while requiring validation, suggests the possibility of multimodal risk assessment integrating clinical factors, echocardiographic, and laboratory parameters.

The predictive value of left atrial enlargement observed in our analysis aligns with emerging concepts of atrial cardiopathy, which is characterized by atrial structural and electrophysiological remodeling and may independently result in a predisposition for thromboembolism beyond AF rhythm alone [15,16,17]. Atrial cardiopathy includes left atrial fibrosis, enlargement, and dysfunction, which create a prothrombotic milieu even in the absence of clinically detected AF episodes. This mechanistic framework provides biological plausibility for why left atrial size predicts visceral embolic events, suggesting that atrial structural abnormalities rather than rhythm disturbances per se may drive thromboembolic risk across all vascular beds. The relationship between atrial cardiopathy biomarkers and non-cerebral embolic events warrants further investigation, particularly as novel markers such as atrial fibrosis indices and NT-proBNP gain clinical traction for risk stratification.

Compared to previous studies in this field identified earlier, our findings provide the first structured synthesis of evidence that has been scattered across previous studies and geographic regions. Prior isolated studies have suggested variable incidence rates and high mortality, but our utilized approach demonstrates the consistency of high mortality burden across different populations while clarifying the sources of incidence variation. Our study extends beyond previous evidence by evaluating treatment effects and identifying novel predictors with significant promising roles.

Despite our study strengths, we have several important limitations that must be acknowledged when interpreting the findings. The significant heterogeneity observed across studies represents a limitation that reflects important differences in study populations, healthcare settings, outcome definitions, and methodologies. This heterogeneity limits the precision of pooled estimates and suggests that results may not be uniformly applicable across all populations and all settings. The heterogeneity arises from several sources, such as geographic and healthcare system differences as Asian vs. Western populations with different diagnostic capabilities and practice settings, temporal variations in 45 years of data along with advancing diagnostic and treatment strategies, and methodological differences in outcome ascertainment ranging from administrative coding to detailed imaging confirmation.

The quality assessment revealed mixed methodological quality across included studies, with only 17% achieving excellent quality ratings and 50% rated as fair quality. Common limitations included inadequate selection of non-exposed cohorts, limited adjustment for confounding variables, insufficient follow-up duration, and possible selection bias toward more severe cases in hospital-based studies. These quality limitations may introduce bias in the pooled estimates and limit the strength of conclusions, especially for the mortality outcomes where hospital-based studies may overestimate case fatality rates due to referral bias toward more severe cases.

The sparse network structure for treatment comparisons precluded formal network meta-analysis and limited our ability to provide structured and detailed comparative effectiveness evidence. With only four direct treatment comparisons available across studies, we could not formulate indirect comparison pathways or rank different therapeutic interventions. This limitation reflects the historical focus on stroke prevention in AF studies and the relative neglect of visceral embolic outcomes.

Publication bias assessment, despite our results suggesting low risk based on statistical tests, was limited by the small number of studies available for each outcome comparison. The visual asymmetry observed in funnel plots and the possible risk for selective publication of positive results cannot be fully excluded. In addition to that, the literature search may have missed studies published in non-English languages or those focusing on specific organ systems without an AF focus.

The outcome definition heterogeneity represents another significant limitation, with studies utilizing different criteria for visceral embolic events ranging from administrative codes to detailed imaging confirmation. This variation may contribute to the observed incidence differences and limits the comparability of results across studies. Some studies focused on specific outcomes such as acute mesenteric ischemia while others utilized composite endpoints, possibly affecting the generalizability of findings.

We successfully addressed significant heterogeneity through systematic subgroup analysis based on event definition. Pooling all prevalence studies together initially resulted in I^2^ = 99.6%, reflecting fundamental differences between AMI events (prevalence 17–52%) and non-AMI visceral events (prevalence 1–5%). By analyzing non-AMI events separately, we achieved I^2^ = 45.4%, representing a 54.2%-point reduction and reclassification from considerable to moderate heterogeneity. This improvement demonstrates that the primary source of heterogeneity was event definition rather than methodological quality or study design. The remaining moderate heterogeneity in the non-AMI subgroup (I^2^ = 45.4%) likely reflects true clinical difference and variability in diagnostic methods (imaging-confirmed versus clinical diagnosis), patient populations (hospital-based versus cross-sectional), and healthcare settings (single-center versus multicenter). Importantly, this I^2^ value is substantially lower than the median I^2^ of 96.9% reported for prevalence meta-analyses in systematic reviews [14], indicating that our subgroup analysis achieved meaningful homogeneity improvement while preserving clinical interpretability. Sensitivity analyses restricted to high-quality studies (NOS ≥ 7) confirmed robustness of findings, with comparable effect estimates despite variation in I^2^ values.

Based on our findings and identified limitations, several important recommendations were concluded for future studies, practice settings, and guideline development. Prospective multicenter cohort studies are needed to provide more precise incidence estimates and mortality data using standardized outcome definitions and advanced diagnostic approaches. These studies should utilize more unified imaging protocols for diagnosis confirmation, standardized follow-up procedures, and consistent data collection methods to minimize the heterogeneity that limits current evidence synthesis. Priority should be given to population-based studies that can provide unbiased incidence estimates free from the selection bias inherent in hospital-based studies.

The development and validation of better risk predictive models integrating novel predictors represents a high-priority study area with significant benefits. Our feasibility assessment demonstrates that a CHA_2_DS_2_-VASc-LAE score integrating left atrial enlargement could improve discriminative ability from C-statistic 0.56 to 0.65–0.70, representing a clinically meaningful improvement. Future studies should focus on prospective derivation and external validation of such improved models, with attention to calibration across different populations and healthcare settings. The inclusion and integration of biomarkers, advanced echocardiographic parameters, and possible artificial intelligence applications could lead to improved predictive performance.

Dedicated randomized controlled trials evaluating anticoagulation strategies for visceral embolic prevention are needed to formulate more accurate evidence-based treatment recommendations. While our study provides supportive evidence for anticoagulation efficacy, the current evidence base relies heavily on observational data and post hoc analyses of stroke prevention trials. Future studies should include visceral embolic events as primary endpoints or secondary endpoints, compare different anticoagulation strategies, and better evaluate intensity and duration of therapy.

Standardization of outcome definitions and diagnostic criteria for visceral embolic events should be formulated through consensus guidelines including relevant medical specialties. This standardization should include clear diagnostic criteria, recommended imaging protocols, severity grading systems, and more structured reporting formats to facilitate future evidence synthesis and quality improvement initiatives. Professional societies and organizations should collaborate to develop position statements on recognition, diagnosis, and management of visceral embolic events in AF.

Healthcare system interventions to improve recognition and early diagnosis of visceral embolic events should be prioritized given the high mortality burden and the possibility for improved outcomes with early intervention. This may include development of clinical decision support tools, emergency department protocols for AF patients presenting with abdominal or limb symptoms, and educational initiatives for healthcare providers regarding the presentation and diagnostic approach for these events.

## 5. Limitations

This study has several limitations. First, significant heterogeneity was observed across studies (I^2^ > 85% for incidence and mortality), reflecting differences in populations, healthcare settings, outcome definitions, and diagnostic methods rather than methodological inconsistency alone. Second, only 17% of studies achieved excellent quality ratings on NOS, with common limitations including inadequate control selection and limited confounding adjustment. Third, the sparse network of only four direct treatment comparisons precluded formal network meta-analysis. Fourth, publication bias assessment was limited by the small number of studies per outcome (k < 10), preventing definitive conclusions. Fifth, outcome definition heterogeneity, with studies using varied terminology (IBD, SEE, NCTE, SDVI, AMI) for visceral events, may affect comparability. Finally, this review was not prospectively registered in PROSPERO. These limitations should be considered when interpreting our findings.

## 6. Conclusions

From our findings, we found that visceral embolic events affect around 1 to 4 patients per 1000 AF patients per year, with mortality rates of 17–74% that significantly exceed those of cerebral stroke. Anticoagulation therapy demonstrates significant protective effects, reducing both mortality and surgical intervention requirements by 50%. The CHA_2_DS_2_-VASc score demonstrated little discriminative ability for visceral embolic prediction of C-statistic 0.56 but maintains utility through a clear 3.6-time risk gradient. Left atrial enlargement was as the strongest novel predictor with 5.12-time increased odds, offering a role for increased risk stratification.

Our findings recommend the integration of visceral embolic considerations into atrial fibrillation management strategies and support extending current anticoagulation guidelines to include visceral embolic prevention. The significant mortality burden and demonstrated treatment efficacy justify prioritizing early recognition, proper early identification and diagnosis, and well selected anticoagulation in certain patients. Improved risk prediction models including left atrial enlargement and other novel predictors should be further tested, validated and developed to improve patient selection for intensive preventive interventions. Dedicated prospective studies and randomized trials focusing on visceral embolic outcomes are needed to formulate high-quality evidence-based management protocols and reduce the significant mortality burden associated with these complications.

## Figures and Tables

**Table 1:** Study characteristics and baseline demographics.

Study	Design	Location	Period	AF Patients (N)	Age Inclusion	AF Type	Follow-Up	Original Outcome Term	VEE Components Extracted	Age (Years)	Female (%)	Diabetes (%)	HTN (%)	HF (%)	CAD (%)	CHA_2_DS_2_-VASc
**Kase et al., 2023** [2]	Retrospective cohort	Helsinki, Finland	2009–2018	577	≥18 years	All types	1 year	AMI (acute mesenteric ischemia)	Mesenteric infarction only	NR	57	NR	81	NR	67	NR
**Liao et al., 2022** [3]	Retrospective cohort	Taiwan (nationwide)	2012–2017	69,549	≥20 years	All types	2.5–3.4 years	IBD (ischemic bowel disease)	Mesenteric ischemia/infarction	75.7 vs. 70.1	NR	38.4	83.5	37.8	11.7	4.20 ± 1.75
**Sohn et al., 2021** [4]	Retrospective cohort	Republic of Korea (single center)	2016–2019	100	≥19 years	All types	In-hospital	SDVI (systemic documented visceral infarction)	Splenic infarction only	73 (median)	NR	29	62	12	NR	5 (4–6)
**Kim et al., 2021** [5]	Retrospective cohort	Republic of Korea (nationwide)	2008–2017	615,724	≥18 years	Newly diagnosed AF	5.9 years (median)	SEE (systemic embolic events)	Mesenteric, splenic, renal infarctions extracted from broader SEE	NR	NR	16.6	55.4	16.7	10.4	NR
**Hu et al., 2017** [6]	Retrospective cohort	Taiwan (nationwide)	2000–2011	212,058	≥20 years	Newly diagnosed AF	2.67 years (mean)	IBD (ischemic bowel disease)	Mesenteric ischemia/infarction	75.6 ± 9.4	51.3	29.2	64.1	36.3	49.7	4.45 ± 1.81
**Weisenburger-Lile et al., 2017** [7]	Retrospective cohort	France (single center)	2014–2016	47	≥65 years	All types	In-hospital	SDVI (systemic documented visceral infarction)	Splenic, renal infarctions	85 (median)	72.3	10.6	72.3	NR	NR	4 (1–8)
**Emren et al., 2017** [8]	Retrospective cohort	Turkey (single center)	2010–2016	115	NR	Non-valvular AF	In-hospital	NCTE (non-cerebral thromboembolism)	Mesenteric, splenic, renal infarctions extracted from broader NCTE	73.4 ± 12.8	53	43	79	25	52	4.29 ± 2.02
**Bhandari et al., 2016** [9]	Cross-sectional	USA (nationwide)	2009–2011	8306	≥18 years	All types	In-hospital	AMI (acute mesenteric ischemia)	Mesenteric infarction only	77.5	NR	3.9	52.3	34.7	14.8	NR
**Christiansen et al., 2016** [10]	Retrospective cohort	Denmark (nationwide)	1997–2011	Population-based	50–89 years	All types	5 years (fixed)	Composite TE (thromboembolism)	Mesenteric, splenic, renal infarctions extracted from composite outcome	Age-stratified	Age-stratified	13–21	41–60	21–41	23–41	Components used
**Bekwelem et al., 2015** [11]	Post hoc RCT analysis	International (RCTs)	~2005–2011	37,973	Varied by trial	AF requiring AC	2.4 years (mean)	SEE (systemic embolic events)	Mesenteric, splenic, renal infarctions extracted from broader SEE	73.1 ± 8.5	56	25.3	81.0	34.5	26.5	4.0 ± 1.5
**Friberg et al., 2012** [12]	Retrospective cohort	Sweden (nationwide)	NR	Large cohort	NR	NR	NR	Composite stroke/TE/bleeding	Mesenteric infarction extracted from composite outcome	NR	NR	~16	~53	~21	~9	Both scores tested
**Hinton et al., 1977** [13]	Autopsy series	UK (single hospital)	1950–1975	333	All ages	All types	NA (autopsy)	Mesenteric embolism	Mesenteric infarction only	NR	NR	NR	32	25	51	NA

Abbreviations: AF = atrial fibrillation; AC = anticoagulation; AMI = acute mesenteric ischemia; CAD = coronary artery disease; HF = heart failure; HTN = hypertension; IBD = ischemic bowel disease NCTE = non-cerebral thromboembolism; NR = not reported; RCT = randomized controlled trial; SDVI = systemic documented visceral infarction; SEE = systemic embolic events; TE = thromboembolism; VEE = visceral embolic events.

**Table 2:** Primary and secondary outcomes analysis.

Outcome Category	Subgroup/Analysis	Studies (k)	Total N/ Person-Years	Individual Study Results	Pooled Estimate	95% CI	I^2^ (%)	τ^2^	Heterogeneity *p*	95% Prediction Interval
**Incidence Rate (per 1000 person-years):**										
VEE Incidence	All studies pooled	3	841,967 PY	Hu 2017 [6]: 3.48 Liao 2022 [3]: 0.36 Bekwelem 2015 [11]: 2.40	**1.46**	0.61–3.51	**99.4**	0.59	<0.001	0.00–121,784
	Taiwan studies only	2	379,495 PY	Hu 2017 [6]: 3.48 Liao 2022 [3]: 0.36	1.12	0.12–10.38	99.6	-	<0.001	-
**Prevalence Non-AMI Visceral Embolic Events:**										
Non-AMI VEE	**Primary analysis**	**3**	**548**	Emren 2017 [8] (NCTE): 5.2% Sohn 2021 [4] (SDVI): 1.0% Hinton 1977 [13] (Mesenteric): 1.2%	**1.6%**	**0.0–3.2%**	**45.4**	**0.0001**	**0.160**	**0.0–7.0%**
	Sensitivity: High quality only (NOS ≥ 7)	2	215	Emren 2017 [8]: 5.2% Sohn 2021 [4]: 1.0%	2.7%	0.0–6.8%	70.3	0.0003	0.067	0.0–17.5%
	Sensitivity: Excluding Emren 2017 [8]	2	433	Sohn 2021 [4]: 1.0% Hinton 1977 [13]: 1.2%	1.1%	0.0–2.5%	0.0	0.00	0.883	-
	Sensitivity: Excluding Sohn 2021 [4]	2	448	Emren 2017 [8]: 5.2% Hinton 1977 [13]: 1.2%	2.4%	0.0–7.6%	71.1	-	0.062	-
	Sensitivity: Excluding Hinton 1977 [13]	2	215	Emren 2017 [8]: 5.2% Sohn 2021 [4]: 1.0%	2.7%	0.0–6.8%	70.3	0.0003	0.067	-
**Prevalence Acute Myocardial Infarction:**										
AMI in AF patients	Both studies	2	8883	Kase 2023 [2]: 52.0% (300/577) Bhandari 2016 [9]: 17.0% (1412/8306)	34.4%	0.1–68.7%	**99.6**	-	<0.001	Not calculated
**In-Hospital Mortality:**										
All in-hospital mortality	All studies pooled	3	8998	Kase 2023 [2]: 64.0% (369/577) Bhandari 2016 [9]: 35.5% (2949/8306) Emren 2017 [8]: 17.4% (20/115)	39.1%	17.6–60.6%	**99.1**	0.67	<0.001	0.0–100%
**Stratified Presentation (Clinically Preferred):**										
High-risk elderly with AMI		1	577	Kase 2023 [2] (Finland, median age 84y)	**64.0%**	60.0–67.9%	-	-	-	-
Mixed nationwide population		1	8306	Bhandari 2016 [9] (USA, age 77.5 ± NR)	**35.5%**	NR	-	-	-	-
Younger cohort, non-AMI VEE		1	115	Emren 2017 [8] (Turkey, age 73.4 ± 12.8)	**17.4%**	11.0–26.0%	-	-	-	-
**Long-term Mortality:**										
30-day mortality (RCT)		1	219	Bekwelem 2015 [11] (International RCT)	**24.7%**	19.1–30.9%	-	-	-	-
1-year mortality		1	577	Kase 2023 [2] (High-risk AMI cohort)	**74.0%**	70.2–77.5%	-	-	-	-

Abbreviations: AF = atrial fibrillation; AMI = acute mesenteric ischemia; CI = confidence interval; I^2^ = I-squared statistic (measure of heterogeneity);; k = number of studies; NCTE = non-cerebral thromboembolism; NOS = Newcastle–Ottawa Scale; NR = not reported; PY = person-years; RCT = randomized controlled trial; SDVI = systemic documented visceral infarction; τ^2^ = tau-squared (between-study variance); VEE = visceral embolic events.

**Table 3:** Risk predictors and clinical factors associated with visceral embolic events in atrial fibrillation.

Study	Analysis Type	Risk Factor/Score	Category/Comparison	Events/Total (n/N)	Event Rate (%)	Effect Measure	Estimate	95% CI	*p*-Value	Original Outcome Term
**CHA_2_DS_2_-VASC Risk Stratification For VEE:**										
Hu et al., 2017 [6]	Risk Score	CHA_2_DS_2_-VASc = 0	Lowest risk	56/15,571	0.36	Reference	-	-	-	IBD (VEE)
		CHA_2_DS_2_-VASc = 1	Low risk	108/21,328	0.51	Rate increase	+0.15%	-	-	
		CHA_2_DS_2_-VASc = 2	Moderate risk	230/34,756	0.66	Rate increase	+0.30%	-	-	
		CHA_2_DS_2_-VASc = 3	Moderate–high risk	363/41,200	0.88	Rate increase	+0.52%	-	-	
		CHA_2_DS_2_-VASc = 4	High risk	439/39,266	1.12	Rate increase	+0.76%	-	-	
		CHA_2_DS_2_-VASc ≥5	Very high risk	767/59,937	1.28	Rate increase	+0.92%	-	-	
**Discriminatory Performance of Risk Scores:**										
Hu et al., 2017 [6]	Discrimination	CHA_2_DS_2_-VASc for VEE	Predicting IBD (VEE)	1963/212,058	0.93	C-statistic	**0.56**	0.55–0.58	-	IBD (VEE)
Friberg et al., 2012 [12]		CHA_2_DS_2_-VASc for stroke	Predicting stroke (reference)	-	-	C-statistic	0.67	0.67–0.68	-	Stroke (comparison)
**Novel Structural/Imaging Predictors:**										
Sohn et al., 2021 [4]	Cardiac Imaging	**Left Atrial Enlargement**	Moderate–severe LA vs. Normal LA	1/15 vs. 0/85	6.7 vs. 0	Adjusted OR	**5.12**	**1.37–19.15**	**0.015**	SDVI (VEE)
Weisenburger-Lile, 2017 [7]		Left Atrial Diameter	Per 1mm increase	-	-	OR	1.08	0.99–1.18	0.09	
**Novel Biomarker Predictors:**										
Kase et al., 2023 [2]	Biomarker	**Lactate level**	Treatment vs. EOLC group	Median: 2.7 vs. 5.3 mmol/L	-	Median difference	−2.6 mmol/L	-	**<0.001**	AMI (VEE subtype)
		**D-dimer level**		Median: 3.7 vs. 4.9 mg/L	-		−1.2 mg/L	-	**0.043**	
**Traditional Clinical Risk Factors:**										
Bhandari et al., 2016 [9]	Clinical Factor	**Female sex**	Female vs. Male	-	-	Crude OR	1.35	NR	NR	AMI (VEE subtype)
		**Peripheral Vascular Disease**	PVD vs. No PVD	-	-		**2.11**	NR	NR	
		**Heart Failure**	HF vs. No HF	-	-		1.31	NR	NR	
		Diabetes Mellitus	DM vs. No DM	-	-		0.57	NR	NR	
		Chronic Kidney Disease	CKD vs. No CKD	-	-		1.09	NR	NR	
**Thyroid Dysfunction (Subgroup Analysis):**										
Kim et al., 2021 [5]	Thyroid Status	Hyperthyroidism	Age <65 years	-	-	Adjusted HR	1.25	1.15–1.36	-	SEE (VEE extracted)
			Age ≥65 years	-	-		1.09	1.03–1.15	-	
**AF-Associated VEE Risk vs. General Population:**										
Christiansen et al., 2016 [10]	Population Risk	AF vs. No AF	Men, age 70y, 0 risk factors	-	13.6 vs. 9.6 per 1000 PY	Rate Ratio	**1.42**	-	-	Composite TE (VEE extracted)
			Women, age 70y, 0 risk factors	-	12.0 vs. 7.0 per 1000 PY		**1.71**	-	-	

Abbreviations: AF = atrial fibrillation; AMI = acute mesenteric ischemia; CI = confidence interval; CKD = chronic kidney disease; DM = diabetes mellitus; EOLC = end-of-life care; ESC = European Society of Cardiology; HF = heart failure; HR = hazard ratio; IBD = ischemic bowel disease; LA = left atrial; LAE = left atrial enlargement; NR = not reported; OR = odds ratio; PVD = peripheral vascular disease; PY = person-years; RR = rate ratio; SDVI = systemic documented visceral infarction; SEE = systemic embolic events; TE = thromboembolism; VEE = visceral embolic events.

**Table 4:** Network meta-analysis and individual patient data simulation.

Study/Analysis Type	Variable/Comparison	Treatment/Category	Control/Reference	Sample Size (N)	Value/Effect	95% CI/SD	*p*-value	Follow-up/Distribution	Original Outcome Term
**Network Meta-Analysis: Available Treatment Comparisons:**									
Liao et al., 2022 [3]	Direct Comparison	IBD incidence	NOAC (44/43787) vs. Warfarin (23/25762)	69,549	aHR: 0.802	0.501–1.342	0.430	2.5–3.4 years	IBD (mesenteric VEE)
Bhandari et al., 2016 [9].	Treatment Effect	In-hospital mortality	AC (148/680) vs. No-AC (2798/7626)	8306	Adj OR: 0.50	0.4–0.6	<0.001	In-hospital	AMI (mesenteric VEE)
Bhandari et al., 2016 [9].	Treatment Effect	Bowel resection	AC (187/680) vs. No-AC (3761/7626)	8306	Adj OR: 0.50	0.4–0.6	<0.001	In-hospital	AMI (mesenteric VEE)
Bekwelem et al., 2015 [11]	Treatment Effect	SEE (AVERROES)	Apixaban vs. Aspirin	37,973	RR: 0.23	0.08–0.64	0.005	2.4 years	SEE (VEE extracted)
**IPD Simulation: Continuous Variables (Mean ± SD):**									
Hu et al., 2017 [6]	Age Distribution	Age (years) IBD group	Population mean	1963	75.6	±9.4	-	Normal distribution	IBD (mesenteric VEE)
Liao et al., 2022 [3]	Age Distribution	Age (years) NOAC group	Population mean	43,787	75.7	NR	-	Normal distribution	IBD (mesenteric VEE)
Hu et al., 2017 [6]	Risk Score	CHA_2_DS_2_-VASc IBD group	Population mean	1963	4.45	±1.81	-	Discrete (0–9)	IBD (mesenteric VEE)
Liao et al., 2022 [3]	Risk Score	CHA_2_DS_2_-VASc NOAC group	Population mean	43,787	4.20	±1.75	-	Discrete (0–9)	IBD (mesenteric VEE)
Bekwelem et al., 2015 [11]	Risk Score	CHA_2_DS_2_-VASc SEE group	Population mean	219	4.0	±1.5	-	Discrete (0–9)	SEE (VEE extracted)
Emren et al., 2017 [8]	Risk Score	CHA_2_DS_2_-VASc NCTE group	Population mean	115	4.29	±2.02	-	Discrete (0–9)	NCTE (VEE extracted)
**IPD Simulation: Comorbidity Prevalences (%):**									
Hu et al., 2017 [6]	Comorbidities	DM/HTN/HF/CAD/CKD IBD	Population prevalence	1963	29.2/64.1/36.3/49.7/9.6	Binomial	-	Binary variables	IBD (mesenteric VEE)
Liao et al., 2022 [3]	Comorbidities	DM/HTN/HF/CAD/CKD NOAC	Population prevalence	43,787	38.4/83.5/37.8/11.7/18.1	Binomial	-	Binary variables	IBD (mesenteric VEE)
Bekwelem et al., 2015 [11]	Comorbidities	DM/HTN/HF/CAD/CKD SEE	Population prevalence	219	25.3/81.0/34.5/26.5/> 80	Binomial	-	Binary variables	SEE (VEE extracted)
Emren et al., 2017 [8]	Comorbidities	DM/HTN/HF/CAD/CKD NCTE	Population prevalence	115	43.0/79.0/25.0/52.0/16.0	Binomial	-	Binary variables	NCTE (VEE extracted)
**Feasibility Assessment:**									
Network Meta-Analysis	Limitation	Network structure	4 direct comparisons	Limited indirect paths	Sparse network	-	Insufficient connections	-	N/A
IPD Simulation	Feasible	Multiple variables	Age CHA_2_DS_2_-VASc comorbidities	Adequate sample sizes ~320,000	Multiple studies	Known distributions	Good foundation	-	N/A
IPD Simulation	Limitation	Correlation structure	Individual correlations	Unknown	Assumption required	-	May affect accuracy	-	N/A
Both Analyses	Required	Additional studies	More direct comparisons	Larger networks	Enhanced precision	-	Future research priority	-	N/A

Abbreviations: AC = Anticoagulation; aHR = adjusted Hazard Ratio; AMI = Acute Mesenteric Ischemia; CAD = Coronary Artery Disease; CHA_2_DS_2_-VASc = CHF, Hypertension, Age ≥ 75 years (2 points), Diabetes, Stroke/TIA/thromboembolism (2 points), Vascular disease, Age 65–74 years, Sex category; CI = Confidence Interval; CKD = Chronic Kidney Disease; DM = Diabetes Mellitus; HF = Heart Failure; HTN = Hypertension; IBD = Ischemic Bowel Disease; IPD = Individual Patient Data; NCTE = Non-Cerebral Thromboembolism; No-AC = No Anticoagulation; NOAC = Novel Oral Anticoagulant; NR = Not Reported; OR = Odds Ratio; RR = Relative Risk; SD = Standard Deviation; SEE = Systemic Embolic Event; VEE = Visceral Embolic Events.

**Table 5:** Sensitivity and subgroup analyses for visceral embolic events meta-analysis.

Analysis Type	Specification	Studies Included (k)	Total N	Pooled Estimate	95% CI	I^2^ (%)	Comparison to Primary	Interpretation
**Primary Analyses:**								
Non-AMI VEE Prevalence	All studies	3	548	1.6%	0.0–3.2%	**45.4**	Reference	**Success: Acceptable heterogeneity**
All VEE Prevalence	Before subgrouping	5	9454	15.2%	-	**99.6**	Baseline	Unacceptable heterogeneity
**Heterogeneity Reduction**	Event definition subgroup	-	-	-	-	**−54.2 points**	99.6% → 45.4%	**Major methodological success**
**Quality-Based Sensitivity Analyses:**								
High Quality Only	NOS ≥ 7, exclude Hinton 1977 [13]	2	215	2.7%	0.0–6.8%	70.3	vs. 1.6% (all)	**Robust**: Similar estimate despite exclusion
Moderate/High Quality	NOS ≥ 6	3	548	1.6%	0.0–3.2%	45.4	Same as primary	All included studies ≥6
Contemporary Studies	2017+ only	2	215	2.7%	0.0–6.8%	70.3	vs. 1.6% (all)	**Robust**: Modern data confirms finding
**Leave-One-Out Sensitivity Analyses:**								
Excluding Emren 2017 [8]	Sohn 2021 [4], Hinton 1977 [13]	2	433	1.1%	0.0–2.5%	**0.0**	vs. 1.6% (all)	**Robust**: I^2^ eliminated, similar estimate
Excluding Sohn 2021 [4]	Emren 2017 [8], Hinton 1977 [13]	2	448	2.4%	0.0–7.6%	71.1	vs. 1.6% (all)	**Robust**: Acceptable I^2^, similar range
Excluding Hinton 1977 [13]	Emren 2017 [8], Sohn 2021 [4]	2	215	2.7%	0.0–6.8%	70.3	vs. 1.6% (all)	**Robust**: Same as high-quality analysis
**Conclusion**	All leave-one-out	-	-	Range: 1.1–2.7%	-	Range: 0–71%	Narrow estimate range	**Not driven by single study**
**Subgroup Analyses By Event Definition:**								
Non-AMI VEE	Mesenteric, splenic, renal	3	548	**1.6%**	0.0–3.2%	**45.4**	Primary analysis	**Acceptable heterogeneity achieved**
AMI Only	Acute mesenteric ischemia	2	8883	34.4%	0.1–68.7%	**99.6**	Cannot pool	Reverse causality issue (Kase 2023)
All VEE Types Mixed	No subgrouping	5	9454	15.2%	-	**99.6**	Baseline unacceptable	**Mixing event types = high I^2^**
**Conclusion**	Event definition primary driver	-	-	-	-	**54.2 pt reduction**	Major finding	**Clinical heterogeneity identified**
**Subgroup Analyses By Study Design:**								
Retrospective Cohorts	Hospital-based	2	215	2.7%	0.0–6.8%	70.3	vs. 1.6% (all)	Acceptable heterogeneity maintained
Including Autopsy	Add historical data	3	548	1.6%	0.0–3.2%	45.4	Primary	Autopsy inclusion improved homogeneity
**Subgroup Analyses By Geographic Region:**								
Asian Studies Only	Turkey, Republic of Korea	2	215	2.7%	0.0–6.8%	70.3	vs. 1.6% (all)	Consistent with overall finding
Including European	Add UK (Hinton 1977 [13])	3	548	1.6%	0.0–3.2%	45.4	Primary	Geographic mixing acceptable
**Subgroup Analyses By Sample Size:**								
Large Studies	n ≥ 100	3	548	1.6%	0.0–3.2%	45.4	Primary (all ≥100)	All studies adequate size
Single-Center	Hospital-based only	2	215	2.7%	0.0–6.8%	70.3	vs. 1.6%	Consistent finding
**Incidence Rate Sensitivity Analyses:**								
All Incidence Studies	3 studies	3	841,967 PY	1.46/1000 PY	0.61–3.51	**99.4**	Primary	**Persistent temporal heterogeneity**
Taiwan Studies Only	Same country	2	379,495 PY	1.12/1000 PY	0.12–10.38	**99.6**	vs. 1.46	**10-fold temporal difference persists**
Contemporary Era	2012+	1	185,572 PY	0.36/1000 PY	0.28–0.46	-	Single study	Lower rate in DOAC era
**Conclusion**	Temporal heterogeneity	-	-	-	-	Cannot reduce	Real change over time	**Evolution of care, not methodology**
**Mortality Sensitivity Analyses:**								
All In-Hospital	3 studies	3	8998	39.1%	17.6–60.6%	**99.1**	Primary	**Persistent population heterogeneity**
Excluding Kase (elderly AMI)	Younger, mixed	2	8421	25.9%	16.8–37.7%	**92.1**	vs. 39.1%	**High I^2^ persists**
Non-AMI Mortality Only	Exclude AMI patients	1	115	17.4%	11.0–26.0%	-	Single study	Lower mortality without AMI
**Conclusion**	Population heterogeneity	-	-	Range: 17–64%	-	Cannot reduce	Real clinical variability	**Age + AMI severity drive variation**

Abbreviations: AMI = acute myocardial infarction; CI = confidence interval; DOAC = direct oral anticoagulant; I^2^ = I-squared statistic (heterogeneity measure); k = number of studies; N = total sample size; NOS = Newcastle–Ottawa Scale; PY = person-years; VEE = visceral embolic events.

**Table 6:** Enhanced Risk Prediction Models From CHA_2_DS_2_-VASC-LAE Score Derivation, Validation Metrics, And Calibration Requirements.

Component/Analysis	Specification	Data Source/Study	Effect Size	95% CI	Points/Weight	Availability	Performance/Requirement	Implementation	Original Outcome Term
**Traditional CHA_2_DS_2_-VASc Performance:**									
Current CHA_2_DS_2_-VASc	VEE prediction (IBD/AMI extracted)	Hu et al. 2017 [6]	C-stat: 0.56	0.55–0.58	0–9 points	Clinical data	Moderate discrimination	Baseline model	IBD (mesenteric VEE)
Risk Gradient	Score stratification	Hu et al. 2017 [6]	0.36–1.28%	Per 100 PY	3.6-fold	212,058 patients	Clear dose-response	Risk stratification	IBD (mesenteric VEE)
**Novel Predictors For Enhanced Model:**									
Left Atrial Enlargement	Moderate-severe LAE	Sohn et al. 2021 [4]	aOR: 5.12	1.37–19.15	+2 points	Echo/MRI	Strongest novel predictor	LAE ≥42 mL/m^2^	SDVI (VEE extracted)
Lactate level	Elevated lactate	Kase et al. 2023 [2]	Strong predictor	*p* < 0.001	+1 point	Laboratory	Prognostic significance	Lactate > 4 mmol/L	AMI (mesenteric VEE)
D-dimer level	Elevated D-dimer	Kase et al. 2023 [2]	Poor prognosis	*p* = 0.043	+1 point	Laboratory	Coagulation marker	D-dimer > 5 mg/L	AMI (mesenteric VEE)
Female sex	Sex-specific risk	Bhandari et al. 2016 [9]	OR: 1.35	NR	+1 point	Clinical	Confirmed predictor	Already in CHA_2_DS_2_-VASc	AMI (mesenteric VEE)
Peripheral Vascular Disease	PVD history	Bhandari et al. 2016 [9]	OR: 2.11	NR	+1 point	Clinical	Strong predictor	Already in CHA_2_DS_2_-VASc	AMI (mesenteric VEE)
**CHA_2_DS_2_-VASc-LAE Enhanced Score Derivation:**									
Traditional Components	C-H-A_2_-D-S_2_-V-A-Sc	Multiple combined	Validated	Established	0–9 points	Clinical	Baseline 0.56 C-statistic	Standard calculation	N/A
Enhanced Components	LAE + Biomarkers	Sohn 2021 [4] Kase 2023 [2]	Strong predictors	Significant	+2–4 points	Echo/Lab	Expected C-stat 0.65–0.70	Additional assessment	N/A
Total Score Range	CHA_2_DS_2_-VASc-LAE-Lab	Combined model	Enhanced model	TBD	0–12 points	Clinical + Tech	Improved discrimination	Comprehensive assessment	N/A
**Internal Validation Specifications:**									
Derivation Dataset	Combined studies	Pooled data	~320,000 patients	~2500 events	227 EPV	Excellent	Adequate for complex model	Split-sample validation	N/A
Bootstrap Validation	Internal validation	Resampling method	1000 bootstraps	Bias-corrected	Standard	Feasible	Optimism adjustment	Bootstrap 95% CI	N/A
Cross-validation	K-fold validation	10-fold recommended	K = 10 folds	Repeated CV	Standard	Feasible	Robust performance estimate	Repeated 10-fold CV	N/A
**External Validation Requirements:**									
Geographic Validation	Western populations	Kase Bekwelem Bhandari	~46,000 patients	Variable events	Feasible	Available	May differ from Asian model	Separate validation cohort	N/A
Temporal Validation	Contemporary cohorts	DOAC era studies	~70,000 patients	Recent events	Feasible	Available	Better calibration expected	Era-specific validation	N/A
Clinical Setting	Hospital vs. Community	Mixed settings	Variable sizes	Setting-specific	Feasible	Available	Baseline risk differences	Setting-stratified analysis	N/A
**Calibration Assessment Methods:**									
Hosmer-Lemeshow Test	Goodness-of-fit	Risk deciles	Chi-square test	*p* > 0.05 good	Standard	Calculable	Overall calibration	10 risk groups	N/A
Calibration Plots	Predicted vs. Observed	Graphical assessment	45° line ideal	Visual inspection	Standard	Implementable	Risk spectrum calibration	Smooth calibration curve	N/A
Calibration Slope	Slope coefficient	Regression method	Slope = 1 ideal	95% CI	Standard	Calculable	Calibration assessment	Logistic regression	N/A
Brier Score	Overall accuracy	Prediction accuracy	0–1 scale	Lower better	Standard	Calculable	Overall performance	Mean squared error	N/A

Abbreviations: LAE = left atrial enlargement; VEE = visceral embolic events; IBD = ischemic bowel disease; AMI = acute mesenteric ischemia; SDVI = splanchnic/mesenteric vascular ischemia; TBD = to be determined; C-stat = concordance statistic; PY = person-years; Aor = adjusted odds ratio; OR = odds ratio; CI = confidence interval; Echo = echocardiography; MRI = magnetic resonance imaging; NR = not reported; PVD = peripheral vascular disease; EPV = events per variable; CV = cross-validation; DOAC = direct oral anticoagulant.

## Data Availability

Applicable data used in our study are found within the main manuscript text and the provided Supplementary Files.

## Supplementary Materials

The following supporting information can be downloaded at: https://www.mdpi.com/article/10.3390/jcm15010188/s1, Checklist S1: PRISMA 2020 Checklist; Table S1: Risk of Bias Assessment Using Newcastle-Ottawa Scale; Table S2: Sensitivity and Leave-One-Out Analyses for Non-AMI Visceral Embolic Event Prevalence; Table S3: GRADE Evidence Quality Assessment.
