# Supplementary material for: Visceral Embolic Events in Atrial Fibrillation: A Systematic Review and Meta-Analysis of Incidence, Mortality, and Risk Prediction
Source: J Clin Med. 2025 Dec 26;15(1):188. doi: 10.3390/jcm15010188 (PMC12786820; doi:10.3390/jcm15010188)
Supplement: Supplementary file 1 [file jcm-15-00188-s001.zip › Supplementary File S1.pdf]

PRISMA 2020 Checklist for Your Systematic Review

Section	Item #	Checklist Item	Reported?	Location in Manuscript	Notes/Assessment
**TITLE**					
**Title**	1	Identify the report as a systematic review	Yes	Title	"Systematic Review and Meta-Analysis" clearly stated in title
**ABSTRACT**					
**Abstract**	2	See the PRISMA 2020 for Abstracts checklist	Yes	Abstract	Structured abstract includes: Introduction, Methods, Results, Conclusions with key details
**INTRODUCTION**					
**Rationale**	3	Describe the rationale for the review in the context of existing knowledge	Yes	Introduction (paragraphs 1-8)	Comprehensive background on AF, thromboembolic events, knowledge gaps clearly articulated
**Objectives**	4	Provide an explicit statement of the objective(s) or question(s) the review addresses	Yes	Introduction (final paragraph)	Five explicit primary objectives listed: (1) incidence rates (2) mortality (3) anticoagulation efficacy (4) risk stratification (5) novel risk factors
**METHODS**					
**Eligibility criteria**	5	Specify the inclusion and exclusion criteria for the review and how studies were grouped for the syntheses	Yes	Methods section 2.1 "Eligibility Criteria"	Detailed inclusion criteria: observational/interventional studies, adult AF patients, visceral embolic outcomes. Exclusion criteria: case reports <10 patients, pediatric, mechanical valves
**Information sources**	6	Specify all databases, registers, websites, organisations, reference lists and other sources searched or consulted to identify studies	Yes	Methods section 2.2 "Search Strategy"	MEDLINE/PubMed, Google Scholar, Cochrane CENTRAL, Web of Science, Scopus. Manual reference list searches. Search date: inception to June 13, 2025
**Search strategy**	7	Present the full search strategies for all databases, registers and websites, including any filters and limits used	Yes	Methods section 2.2 "Search Strategy"	Comprehensive search terms provided including combinations of AF terms, embolic event terms, organ-specific terms, anticoagulation terms, and risk stratification terms
**Selection process**	8	Specify the methods used to decide whether a study met the inclusion criteria of the review	Yes	Methods section 2.1 "Study Selection and Data Extraction"	Two-stage process: title/abstract screening followed by full-text evaluation
**Data collection process**	9	Specify the methods used to collect data from reports, including how many reviewers collected data from each report	Yes	Methods section 2.1 "Study Selection and Data Extraction"	Data extraction process described but number of independent reviewers not explicitly stated
**Data items**	10a	List and define all outcomes for which data were sought. Specify whether all results that were compatible with each outcome domain in each study were sought	Yes	Methods section 2.1 "Study Selection and Data Extraction"	Comprehensive list: study characteristics, demographics, AF characteristics, visceral embolic definitions, outcomes (incidence, mortality, treatment effects), risk factors
	10b	List and define all other variables for which data were sought	Yes	Methods section 2.1 "Study Selection and Data Extraction"	Variables detailed: sample size, age, gender, comorbidities, AF type, anticoagulation status, follow-up duration, quality parameters
**Study risk of bias assessment**	11	Specify the methods used to assess risk of bias in the included studies	Yes	Methods section 2.1 "Risk of Bias Assessment"	Newcastle-Ottawa Scale (NOS) used, evaluating selection, comparability, and outcome ascertainment. Quality scoring defined: 7-9 high, 4-6 moderate, 0-3 low
**Effect measures**	12	Specify for each outcome the effect measure(s) used in the synthesis or presentation of results	Yes	Methods section 2.1 "Statistical Analysis"	OR/RR with 95% CI for dichotomous outcomes, MD/SMD for continuous outcomes, incidence rates per person-years with inverse variance weighting
**Synthesis methods**	13a	Describe the processes used to decide which studies were eligible for each synthesis	Yes	Methods section 2.1 "Statistical Analysis"	Random-effects vs fixed-effects model selection based on heterogeneity ($I^2 \geq 50\%$ or $P<0.10$)
	13b	Describe any methods required to prepare the data for presentation or synthesis	Yes	Methods section 2.1 "Statistical Analysis"	Mantel-Haenszel method for pooling, inverse variance weighting for incidence rates
	13c	Describe any methods used to tabulate or visually display results of individual studies and syntheses	Yes	Results section	Forest plots, funnel plots, Cleveland dot plots described and presented
	13d	Describe any methods used to synthesize results and provide a rationale for the choice(s)	Yes	Methods section 2.1 "Statistical Analysis"	DerSimonian-Laird random-effects model, meta-analysis using metafor package in R 4.4.2
	13e	Describe any methods used to explore possible causes of heterogeneity among study results	Yes	Methods section 2.1 "Statistical Analysis"	Subgroup analyses by: study design, geographic region, patient population (hospital vs population-based), anticoagulation status, outcome definition
	13f	Describe any sensitivity analyses conducted to assess robustness of the synthesized results	Yes	Methods section 2.1 "Statistical Analysis"	Sensitivity analyses: excluding high risk of bias studies, small studies (<100 participants), studies contributing to significant heterogeneity
**Reporting bias assessment**	14	Describe any methods used to assess risk of bias due to missing results in a synthesis	Yes	Methods section 2.1 "Statistical Analysis"	Funnel plots, Egger's linear regression test, Begg's rank correlation test, trim-and-fill adjustment method
**Certainty assessment**	15	Describe any methods used to assess certainty (or confidence) in the body of evidence for an outcome	Partial	Methods section 2.1 "Statistical Analysis"	Statistical power calculations provided but formal GRADE assessment not explicitly mentioned
**RESULTS**					
**Study selection**	16a	Describe the results of the search and selection process, from the number of records identified in the search to the number of studies included in the review	Yes	Results section 3.1 + Figure 1	PRISMA flowchart: 243 database records + 5 other sources → 101 screened → 38 full-text assessed → 12 included
	16b	Cite studies that might appear to meet the inclusion criteria, but which were excluded, and explain why they were excluded	Yes	Results section 3.1	Numbers provided
**Study characteristics**	17	Cite each included study and present its characteristics	Yes	Results section 3.1 + Table 1	Table 1 provides comprehensive characteristics: author, year, country, design, sample size, demographics, comorbidities, CHA_2DS_2-VASc scores
**Risk of bias in studies**	18	Present assessments of risk of bias for each included study	Yes	Results section 3.3 + Table 1	NOS quality scores presented: 2 excellent (8-9 points), 3 good (6-7), 6 fair (4-5), 1 poor (3 points). Mean score 5.8/9
**Results of individual studies**	19	For all outcomes, present, for each study: (a) summary statistics for each group (where appropriate) and (b) an effect estimate and its precision	Yes	Results Tables 2-6	Comprehensive outcome data: incidence rates, mortality rates, treatment effects with 95% CI, p-values for all studies
**Results of syntheses**	20a	For each synthesis, briefly summarise the characteristics and risk of bias among contributing studies	Yes	Results section 3.4-3.15	Each outcome synthesis includes heterogeneity assessment, quality considerations, and study characteristics
	20b	Present results of all statistical syntheses conducted	Yes	Results Tables 2-6	All planned syntheses presented: incidence, mortality, treatment effects, CHA_2DS_2-VASc performance, novel predictors
	20c	Present results of all investigations of possible causes of heterogeneity among study results	Yes	Results Table 5	Heterogeneity assessment with I^2 statistics, planned meta-regression covariates, subgroup analyses limitations discussed
	20d	Present results of all sensitivity analyses conducted to assess the robustness of the synthesized results	Yes	Results section 3.6 + Table 5	Sensitivity analyses described, power calculations provided showing >95% power for primary outcomes
**Reporting biases**	21	Present assessments of risk of bias due to missing results for each synthesis assessed	Yes	Results section 3.15 + Figures 3-4	Funnel plot visual inspection, asymmetric distribution noted, trim-and-fill adjustment mentioned, limitations due to small study numbers acknowledged
**Certainty of evidence**	22	Present assessments of certainty (or confidence) in the body of evidence for each outcome assessed	Partial	Discussion section	Certainty discussed narratively (high heterogeneity, mortality burden consistent, etc.) but formal GRADE assessment not explicitly presented

**DISCUSSION**					
**Discussion**	23a	Provide a general interpretation of the results in the context of other evidence	Yes	Discussion paragraphs 1-6	Results interpreted in context of AF management, thromboembolic complications, comparison with cerebral stroke, implications for guidelines
	23b	Discuss any limitations of the evidence included in the review	Yes	Discussion + Results sections	Heterogeneity discussed, methodological quality noted (mean 5.8/9)
	23c	Discuss any limitations of the review processes used	Yes	Scattered throughout	Network meta-analysis limitations noted, publication bias concerns mentioned
	23d	Discuss implications of the results for practice, policy, and future research	Yes	Discussion paragraphs 7-12	Extensive discussion: clinical implications (mortality burden, anticoagulation benefits), guideline implications, need for enhanced risk models, future research directions
**OTHER INFORMATION**					
**Registration and protocol**	24a	Provide registration information for the review, including register name and registration number	No	Not mentioned	No registration information provided (common gap in many reviews)
	24b	Indicate where the review protocol can be accessed	No	Not mentioned	Protocol access not mentioned
	24c	Describe and explain any amendments to information provided at registration or in the protocol	No	Not mentioned	N/A if not registered
**Support**	25	Describe sources of financial or non-financial support for the review	Yes	Check funding section	Yes
**Competing interests**	26	Declare any competing interests of review authors	Yes	Check declarations	Yes
**Availability of data, code and other materials**	27	Report which of the following are publicly available and where they can be found: study protocol, extracted data, code used in analyses, other materials used in the review	Yes	Author Contributions section	"Data Availability: Applicable data used in our study are found within main manuscript text and the provided supplementary files."
