# Supplementary material for: Visceral Embolic Events in Atrial Fibrillation: A Systematic Review and Meta-Analysis of Incidence, Mortality, and Risk Prediction
Source: J Clin Med. 2025 Dec 26;15(1):188. doi: 10.3390/jcm15010188 (PMC12786820; doi:10.3390/jcm15010188)
Supplement: Supplementary file 1 [file jcm-15-00188-s001.zip › Supplementary Table S1.pdf]

Supplementary Table S1: Risk of Bias Assessment Using Newcastle-Ottawa Scale.

Study	Design	Country	Outcome	SELECTION				COMPARABILITY		OUTCOME			Total	Quality
				**S1**	**S2**	**S3**	**S4**	**C1**	**C2**	**O1**	**O2**	**O3**	**/9**	**Rating**
Christiansen et al. 2016	Prospective cohort	Denmark	Incidence	★	★	★	★	★	★	★	★	★	**9**	**Excellent**
Bekwelem et al. 2015	RCT post-hoc	International	Incidence/Mortality	★	★	★	★	★	★	★	★	★	**9**	**Excellent**
Kim et al. 2021	Retrospective cohort	South Korea	Prevalence	★	★	★	★	★	★	★	★	☆	**8**	**Good**
Bhandari et al. 2016	Retrospective cohort	USA	Prevalence/Mortality	★	★	★	★	★	☆	★	★	☆	**7**	**Good**
Liao et al. 2022	Retrospective cohort	Taiwan	Incidence	★	★	★	★	★	★	★	★	☆	**7**	**Good**
Kase et al. 2022	Retrospective cohort	Finland	Prevalence/Mortality	★	★	★	★	★	☆	★	★	☆	**7**	**Good**
Emren et al. 2017	Retrospective cohort	Turkey	Prevalence/Mortality	★	★	★	★	★	☆	★	☆	☆	**6**	**Good**
Hu et al. 2017	Retrospective cohort	Taiwan	Incidence	★	★	★	★	★	☆	★	★	☆	**7**	**Good**
Sohn et al. 2021	Cross-sectional	South Korea	Prevalence	★	☆	★	★	★	☆	★	☆	☆	**6**	**Good**
Weisenburger-Lile et al. 2017	Retrospective cohort	France	Prevalence	★	★	★	☆	☆	☆	★	☆	☆	**5**	**Fair**
Friberg et al. 2012	Retrospective cohort	Sweden	Incidence	★	★	★	☆	☆	☆	★	☆	☆	**5**	**Fair**
Hinton et al. 1977	Autopsy series	UK	Prevalence	☆	★	★	☆	☆	☆	★	☆	☆	**4**	**Fair**

NOS Criteria (max 9★): *Selection (4★): S1=representativeness, S2=cohort selection, S3=exposure ascertainment, S4=baseline outcome; Comparability (2★): C1=main confounders, C2=additional confounders; Outcome (3★): O1=assessment, O2=follow-up duration, O3=follow-up adequacy. Quality: Excellent 8-9★, Good 6-7★, Fair 4-5★, Poor 0-3★. Studies (n=12): mean 6.7±1.6, median 7, range 4-9; 2 Excellent (16.7%), 7 Good (58.3%), 3 Fair (25.0%). ★=met, ☆=not met.*
