# Supplementary material for: Visceral Embolic Events in Atrial Fibrillation: A Systematic Review and Meta-Analysis of Incidence, Mortality, and Risk Prediction
Source: J Clin Med. 2025 Dec 26;15(1):188. doi: 10.3390/jcm15010188 (PMC12786820; doi:10.3390/jcm15010188)
Supplement: Supplementary file 1 [file jcm-15-00188-s001.zip › Supplementary Table S2.pdf]

Supplementary Table S2: Sensitivity And Leave-One-Out Analyses For Non-Ami Visceral Embolic Event Prevalence.

Analysis Type	Studies Included	Studies Excluded	Number of Studies (k)	Total Sample Size (n)	Total Events	Pooled Prevalence (%)	95% CI Lower (%)	95% CI Upper (%)	I² (%)	τ²	P-value (Heterogeneity)	Interpretation
**PRIMARY ANALYSIS:**												
Main Analysis (Non-AMI VEE)	Emren 2017; Sohn 2021; Hinton 1977	None	3	548	11	1.6	0.0	3.2	45.4	0.0001	0.160	Moderate heterogeneity - acceptable for meta-analysis
**QUALITY-BASED SENSITIVITY ANALYSES:**												
High-Quality Only (NOS≥7)	Emren 2017 (NOS=6); Sohn 2021 (NOS=6)	Hinton 1977 (NOS=5)	2	215	7	2.7	0.0	6.8	70.3	0.0012	0.067	Comparable to main analysis - findings robust
Contemporary Studies (≥2017)	Emren 2017; Sohn 2021	Hinton 1977 (1977)	2	215	7	2.7	0.0	6.8	70.3	0.0012	0.067	Same as high-quality analysis
**LEAVE-ONE-OUT SENSITIVITY ANALYSES:**												
Excluding Emren 2017	Sohn 2021; Hinton 1977	Emren 2017	2	433	5	1.1	0.0	2.7	0.0	0.0000	0.885	Zero heterogeneity - highly consistent
Excluding Sohn 2021	Emren 2017; Hinton 1977	Sohn 2021	2	448	10	2.4	0.0	6.0	71.1	0.0013	0.062	Moderate-high heterogeneity - acceptable
Excluding Hinton 1977	Emren 2017; Sohn 2021	Hinton 1977	2	215	7	2.7	0.0	6.8	70.3	0.0012	0.067	Moderate-high heterogeneity - acceptable
**SUBGROUP BY STUDY DESIGN:**												
Retrospective Cohorts Only	Emren 2017; Sohn 2021	Hinton 1977 (autopsy)	2	215	7	2.7	0.0	6.8	70.3	0.0012	0.067	Consistent with main analysis
Including Autopsy Study	All three studies	None	3	548	11	1.6	0.0	3.2	45.4	0.0001	0.160	Autopsy inclusion reduces heterogeneity
**SUBGROUP BY GEOGRAPHIC REGION:**												
Asian Studies Only	Emren 2017 (Turkey); Sohn 2021 (South Korea)	Hinton 1977 (UK)	2	215	7	2.7	0.0	6.8	70.3	0.0012	0.067	Similar to quality-based analysis
Including European Study	All three studies	None	3	548	11	1.6	0.0	3.2	45.4	0.0001	0.160	Geographic diversity reduces heterogeneity
**SUBGROUP BY SAMPLE SIZE:**												
Large Studies (n≥100)	Emren 2017 (n=115); Sohn 2021 (n=100); Hinton 1977 (n=333)	None	3	548	11	1.6	0.0	3.2	45.4	0.0001	0.160	All studies have adequate sample size
Single-Center Studies	Emren 2017; Sohn 2021	Hinton 1977 (autopsy series)	2	215	7	2.7	0.0	6.8	70.3	0.0012	0.067	Setting does not drive heterogeneity
**HETEROGENEITY REDUCTION ANALYSIS:**												
Before Event-Definition Subgrouping	All 5 prevalence studies (including AMI)	None	5	9,431	Variable	15.2	NA	NA	99.6	High	<0.001	Unacceptable heterogeneity - pooling inappropriate
After Non-AMI Subgrouping	Non-AMI studies only (Emren; Sohn; Hinton)	AMI studies excluded	3	548	11	1.6	0.0	3.2	45.4	0.0001	0.160	Heterogeneity reduced by 54.2 percentage points
**RANGE OF ESTIMATES ACROSS SENSITIVITY ANALYSES:**												
Minimum Estimate	Sohn + Hinton (excluding Emren)	Emren 2017	2	433	5	1.1	0.0	2.7	0.0	0.0000	0.885	Lower bound of sensitivity range
Maximum Estimate	Emren + Sohn (excluding Hinton)	Hinton 1977	2	215	7	2.7	0.0	6.8	70.3	0.0012	0.067	Upper bound of sensitivity range
Range Summary	Across all sensitivity analyses	Variable	2-3	215-548	5-11	1.1-2.7	NA	NA	0.0-71.1	Variable	Variable	All estimates within narrow range (1.1-2.7%), confirming robustness

Abbreviations: *AMI = Acute Mesenteric Ischemia; CI = Confidence Interval; I² = I-squared heterogeneity statistic; k = Number of studies; n = Sample size; NA = Not Applicable; NOS = Newcastle-Ottawa Scale; τ² = Tau-squared (between-study variance); VEE = Visceral Embolic Events.*
