# Supplementary material for: Visceral Embolic Events in Atrial Fibrillation: A Systematic Review and Meta-Analysis of Incidence, Mortality, and Risk Prediction
Source: J Clin Med. 2025 Dec 26;15(1):188. doi: 10.3390/jcm15010188 (PMC12786820; doi:10.3390/jcm15010188)
Supplement: Supplementary file 1 [file jcm-15-00188-s001.zip › Supplementary Table S3.pdf]

Supplementary Table S3: GRADE Evidence Quality Assessment.

**Outcome**	**Number of Studies (Participants)**	**Study Design**	**Risk of Bias**	**Inconsistency**	**Indirectness**	**Imprecision**	**Publication Bias**	**Effect Size Magnitude**	**Dose-Response Gradient**	**Residual Confounding**	**Starting Quality**	**Quality Rating Adjustments**	**Final Quality of Evidence**	**Comments**
**PRIMARY OUTCOMES:**														
Non-AMI VEE Prevalence	3 studies (n=548)	Observational cohorts	Not serious (NOS 5-6)	Not serious (I^2=45.4%)	Not serious (direct AF populations)	Serious (wide CI: 0.0-3.2%, crosses clinically important thresholds)	Undetected (k<10, limited assessment)	Not applicable	Not applicable	Unlikely to change estimate	LOW	-1 (imprecision)	**LOW** ⊕⊕○○	Moderate heterogeneity acceptable; precision limited by small event numbers (n=11 events)
In-Hospital Mortality (Non-AMI populations)	2 studies (n=8,421)	Observational cohorts	Not serious (NOS 6-7)	Very serious (I^2=99.1%, 17.4% vs 35.5%)	Not serious (direct VEE populations)	Not serious (narrow CIs, large sample)	Undetected (k<10)	Large effect (mortality 17.4-35.5%)	Not applicable	Present but accounted for (age, comorbidities adjusted)	LOW	-2 (inconsistency), +1 (large effect)	**VERY LOW** ⊕○○○	Extreme heterogeneity reflects genuine population differences; stratified presentation appropriate; not pooled
In-Hospital Mortality (Elderly+AMI)	1 study (n=577)	Observational cohort	Not serious (NOS 8)	Not assessable (single study)	Not serious (specific high-risk population)	Not serious (narrow CI: 60.0-67.9%)	Not assessable (single study)	Very large effect (64.0% mortality)	Not applicable	Present but adjusted	LOW	+1 (very large effect)	**LOW** ⊕⊕○○	Single high-quality study; effect magnitude compelling despite observational design
Extended Mortality (30-day to 1-year)	2 studies (n=796)	Observational (1 RCT post-hoc)	Not serious (NOS 8-9)	Serious (I^2=75%, different timeframes)	Not serious (direct VEE populations)	Not serious (adequate precision)	Undetected (k=2)	Large effect (24.7-74.0%)	Not applicable	Minimal (RCT-derived data)	MODERATE	-1 (inconsistency), +1 (large effect)	**MODERATE** ⊕⊕⊕○	RCT-derived data elevates starting quality; heterogeneity from different follow-up durations
**TREATMENT EFFECTS:**														
Anticoagulation vs No Anticoagulation (Mortality + Bowel Resection)	1 study (n=8,306)	Observational cohort	Not serious (NOS 7, adjusted analysis)	Not assessable (single study)	Not serious (direct comparison)	Not serious (narrow CI: 0.4-0.6)	Not assessable (single study)	Large effect (OR 0.50, 50% reduction)	Not applicable	Well-controlled (multivariable adjustment)	LOW	+1 (large effect)	**LOW** ⊕⊕○○	Large nationwide database; robust multivariable adjustment; consistent benefit across outcomes
NOAC vs Warfarin (IBD incidence)	1 study (n=615,724)	Observational cohort	Not serious (NOS 7, propensity-matched)	Not assessable (single study)	Not serious (direct comparison)	Serious (wide CI: 0.50-1.34, crosses null)	Not assessable (single study)	Not applicable (null finding)	Not applicable	Minimal (propensity matching)	LOW	-1 (imprecision)	**VERY LOW** ⊕○○○	Very large sample but few events; precision insufficient to detect moderate effects; propensity matching reduces bias
Apixaban vs Aspirin (Systemic Embolic Events)	1 study (n=5,599)	RCT (AVERROES trial)	Not serious (low RoB, double-blind RCT)	Not assessable (single study)	Serious (SEE as proxy for VEE, not direct VEE outcome)	Not serious (narrow CI: 0.08-0.64)	Not assessable (single study)	Very large effect (RR 0.23, 77% reduction)	Not applicable	None (randomization)	HIGH	-1 (indirectness), +2 (very large effect)	**HIGH** ⊕⊕⊕⊕	RCT evidence with dramatic effect; indirectness offset by magnitude; best available anticoagulation evidence
**RISK PREDICTION:**														
CHA_2DS_2-VASc Score (VEE prediction)	1 study (n=212,058)	Observational cohort	Not serious (NOS 7, large registry)	Not assessable (single study)	Not serious (direct VEE outcome)	Not serious (narrow CIs, 1,963 events)	Not assessable (single study)	Moderate discrimination (C-stat 0.56)	Present (3.6-fold gradient, linear R^2=0.993)	Well-adjusted	LOW	+1 (dose-response)	**LOW** ⊕⊕○○	Clear dose-response despite modest discrimination; clinical utility preserved; very large sample with adequate events
CHA_2DS_2-VASc Score (Stroke prediction, comparator)	1 study (n=182,678)	Observational cohort	Not serious (NOS 7)	Not assessable (single study)	Serious (stroke not VEE)	Not serious (C-stat 0.67, narrow CI)	Not assessable (single study)	Better discrimination for stroke than VEE	Not assessed	Standard application	LOW	-1 (indirectness)	**VERY LOW** ⊕○○○	Included for comparison only; demonstrates CHA_2DS_2-VASc designed for stroke, not visceral events
**NOVEL PREDICTORS:**														
Left Atrial Enlargement (Moderate-to-Severe)	1 study (n=100)	Observational cohort	Serious (NOS 6, small single-center)	Not assessable (single study)	Not serious (direct VEE outcome)	Serious (wide CI: 1.37-19.15, small sample)	Not assessable (single study)	Very large effect (aOR 5.12, 5-fold increase)	Not applicable	Well-adjusted (multivariable)	LOW	-1 (risk of bias), -1 (imprecision), +2 (very large effect)	**LOW** ⊕⊕○○	Strongest single predictor identified; compelling effect size overcomes sample size limitations; requires external validation
Elevated Lactate Levels	1 study (n=577)	Observational cohort	Not serious (NOS 8)	Not assessable (single study)	Not serious (direct VEE population)	Not serious (p<0.001)	Not assessable (single study)	Large difference (median -2.6 mmol/L)	Not applicable	Present (disease severity marker)	LOW	+1 (large effect)	**LOW** ⊕⊕○○	Strong prognostic marker; consistent with ischemia pathophysiology; confounded by disease severity
Elevated D-dimer Levels	1 study (n=577)	Observational cohort	Not serious (NOS 8)	Not assessable (single study)	Not serious (direct VEE population)	Not serious (p=0.043)	Not assessable (single study)	Moderate difference (median -1.2 mg/L)	Not applicable	Present (thrombosis marker)	LOW	No adjustment	**LOW** ⊕⊕○○	Consistent with thrombotic mechanism; less specific than lactate; confounded by acute thrombotic state

GRADE Quality Levels: ***⊕⊕⊕⊕ HIGH:*** *Very confident that the true effect lies close to the estimate of the effect;* ***⊕⊕⊕○ MODERATE:*** *Moderately confident in the effect estimate; true effect likely close to estimate but possibility it is substantially different;* ***⊕⊕○○ LOW:*** *Confidence in effect estimate is limited; true effect may be substantially different from estimate;* ***⊕○○○ VERY LOW:*** *Very little confidence in the effect estimate; true effect likely substantially different from estimate.* ***Abbreviations:*** *aOR = adjusted Odds Ratio; AF = Atrial Fibrillation; AMI = Acute Mesenteric Ischemia; CI = Confidence Interval; C-stat = C-statistic; CHA_2DS_2-VASc = CHF, Hypertension, Age ≥75 years (2 points), Diabetes, Stroke/TIA/thromboembolism (2 points), Vascular disease, Age 65-74 years, Sex category; GRADE = Grading of Recommendations Assessment, Development and Evaluation; HR = Hazard Ratio; I^2 = I-squared heterogeneity statistic; IBD = Ischemic Bowel Disease; k = Number of studies; n = Sample size; NOAC = Novel Oral Anticoagulant; NOS = Newcastle-Ottawa Scale; OR = Odds Ratio; PICO = Population, Intervention, Comparison, Outcome; RCT = Randomized Controlled Trial; RoB = Risk of Bias; RR = Relative Risk; SEE = Systemic Embolic Event; VEE = Visceral Embolic Events.*
